# Enhanced Oxygen Evolution Reaction Activity of a Cerium Oxide-Modified Lanthanum Manganese Oxide Perovskite Catalyst in an Anion Exchange Membrane Water Electrolyzer

**DOI:** 10.1021/acs.energyfuels.5c03080

**Published:** 2025-10-14

**Authors:** Masoud Nouri, Jian Huang, Shanmugam Ramakrishnan, Gaurav Gupta, Stevin Pramana, Mohamed Mamlouk

**Affiliations:** † School of Engineering, 5994 Newcastle University, Newcastle upon Tyne, NE1 7RU, U.K.; ‡ School of Engineering, 4396 Lancaster University, Lancaster, LA1 4YW, U.K.

## Abstract

There has been significant interest in the development of oxygen evolution reaction (OER) catalysts without the use of precious metals, in order to reduce the cost of electrolyzers for green hydrogen production. Herein, different weights (up to 20%) of CeO_2_ added to lanthanum manganese perovskite oxide were synthesized by using the sol–gel method, followed by calcination at 900 °C in air. The phase purity of the prepared CeO_2_–lanthanum manganese perovskite oxide electrocatalysts were investigated by using X-ray diffraction (XRD), followed by Rietveld analysis. The results confirmed that only perovskite and CeO_2_ phases were present without extra impurity phases. An iodometric titration technique was employed to determine the chemical formula and the average oxidation state of Mn in the prepared electrocatalysts. The optimized electrocatalyst containing ∼10 wt % of CeO_2_ content with lanthanum manganese perovskite (LCM-0.1) showed improved OER activity, achieving a greater than 22-fold increase in generated current density at 1.9 V versus RHE (reversible hydrogen electrode) in 0.1 M KOH compared to pure LaMnO_3_. The electrocatalysts were tracked via in-operando Raman spectroscopy and ex-situ XPS spectroscopy, which evidenced reconstruction of the catalyst surface. The seen electrocatalytic activity improvement has been attributed to restructuring of the catalyst surface to form the MnOOH structure. A water electrolyzer was fabricated using the optimized LCM-0.1 electrocatalyst, and the device performance was evaluated with different loadings of the LCM-0.1 catalyst in the anode. The results suggest that the incorporation of cerium oxide in perovskite-based catalysts can be utilized as a method to promote OER electrocatalytic activity.

## Graphical abstract

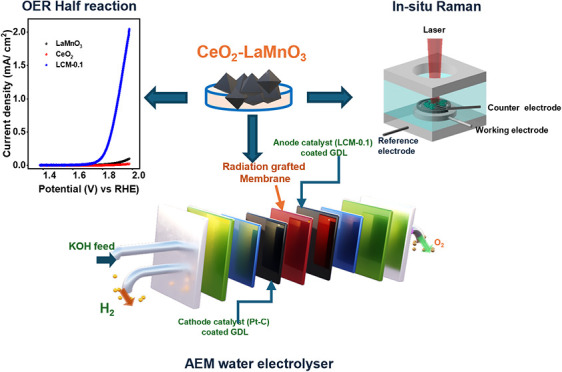

## 1 Introduction

Energy insecurity and pollution have created a demand for alternative energy technologies that are environmentally benign and offer high energy densities. Increasing global energy demand, the scarcity of fossil fuels, and the urgent challenges of climate change are driving the research community to explore sustainable and clean energy technologies. Hydrogen is a potential energy carrier as compared to fossil fuels due to its high energy density, zero carbon emissions, and abundant availability of water as a raw material.^1,2^ Electrochemical water splitting has emerged as a highly promising method for hydrogen production; however, the efficiency of an electrolyzer device depends on oxygen evolution and hydrogen evolution reactions at the anode and cathode, respectively.^3^ The kinetics of the OER is generally sluggish, as it involves a four-electron transfer process per mole of oxygen, and typically, an expensive state-of-the-art IrO_2_/RuO_2_ catalyst is commonly used to accelerate the OER. However, the scarcity of IrO_2_/RuO_2_ is a cause of concern for the scale-up and large deployment of water electrolyzer technology. Therefore, it is necessary to yield economically affordable, earth-abundant, and resilient non-precious OER electrocatalysts. Recently, the development of perovskite oxide-based OER electrocatalysts for water splitting in alkaline media and anion exchange membrane water electrolyzers has gained significant attention due to their tunable electronic properties that can be achieved through the introduction of various dopants, resulting in controlled oxygen vacancies. Perovskite oxides, chemically described as ABO_3_, where the A lattice site is occupied by rare earth and alkaline earth metals, while B typically represents transition metals, e.g., Ba_0.5_Sr_0.5_Co_0.8_Fe_0.2_O_3‑δ_ (BSCF),^4^ have been a center of attention as potential candidates to be used as an efficient OER catalysts in alkaline media. A number of performance descriptors have been proposed to explain the activity of perovskite oxides,^4,5^ suggesting that the number of electrons in the d-orbital of the transition metal of the B-site element, as well as the covalency between the B-site ion and oxygen, determines the activity.

It is widely accepted that the OER process in alkaline media can be explained by different mechanisms, among which the absorbate evolving mechanism and/or lattice oxygen mechanism (LOM) are the two commonly proposed reaction pathways.^3,6,7^ The former involves surface metal-ion sites serving as the catalytic center for reacting molecules to generate oxygen through a four concerted proton electron transfer (CPET). Density functional theory (DFT) suggested that OER catalysts undergoing the LOM route had the potential to deliver higher electrocatalytic output compared to the adsorbate evolution mechanism.^8^

LOM delivers high catalytic activity due to the direct participation of lattice oxygen in the OER, which enhances reaction kinetics.^9,10^ In contrast, the adsorbate evolving mechanism involves the reaction proceeding through adsorbed intermediates such as hydroxyl groups and peroxo species. Therefore, LOM avoids these intermediate steps; as a result, it has faster and more efficient catalytic performance.^8^ Furthermore, mass spectroscopy and isotope labeling have provided experimental footprints that oxygen could be generated from the lattice oxygen in perovskite oxides.^11^ Following the emergence of perovskite oxides as electrocatalytic materials, several strategies for enhancing the performance have been proposed from using a dopant and simultaneously incorporating oxygen vacancies^3^ to increasing the electrochemical activity in energy conversion devices.^12^ Therefore, lanthanum-based perovskite oxides (LaBO_3_) with B-sites of Fe, Co, Ni, and Mn are seen as promising OER electrocatalysts presently.^9,13^ Among the fundamentally studied undoped perovskites, LaMnO_3_ was reported to show a current density of 5 × 10^–8^ A/cm^2^ at an overpotential of 0.3 V (1.53 V vs RHE at room temperature in a 1 M NaOH solution), while 1.3 × 10^–5^ and 2.9 × 10^–6^ A/cm^2^ were obtained for LaNiO_3_ and LaCoO_3_, respectively, under the same conditions.^14^ Altervalent dopants that are introduced into the A-site, which is occupied by the host trivalent metal cation, lead to a charge imbalance. This is mitigated by either the creation of oxygen vacancies or the change in the transition metal’s oxidation state. Most of the transition metals have this capability to adapt and maintain charge neutrality, while if the oxygen vacancies are created, it usually proceeds according to the equation ( $2M^{n+}O\overset{F^{\left(n-1\right)+}\;O}{\to }2M_{F}^{\prime }+V_{O}^{..}+2O_{o}^{\times })$ shown in the Kröger–Vink notation for a divalent substitution (*M* = host ion, *F* = dopant ion, ′ = single negative, ˙ = single positive, and × = neutral).^15^ Contrary to the extensive research in literature on doping strategies,^16^ an increasing number of studies have shown that the performance of the catalyst is not consistent with the volcano plot (plotted against the number of e_g_ electrons), and many studies have revealed that the catalyst structure will change, in particular at the surface. New active sites will be formed when the catalytic process begins.^17,18^ For example, Fabbri et al.^17^ revealed that a dynamic self-reconstruction behavior happened on the Ba_0.5_Sr_0.5_Co_0.8_Fe_0.2_O_3−δ_ surface during OER. A self-constructed metal oxyhydroxide active surface layer structure was formed when the applied potential reached above 1.425 V versus RHE (reversible hydrogen electrode), with the newly formed active layer, leading to higher catalytic performance. This reconstruction mechanism obviously results in a new structure that will not follow the generally accepted relationship between the OER performance and the static catalyst structure and thus provides a new perspective for future catalyst design.^17^ The reconstruction was also reported for a MnO_2_-based catalyst, albeit affecting the bulk structure too.^19^

Most catalyst studies are typically conducted under highly alkaline conditions, such as in 1 M KOH solutions. However, in anion exchange membrane (AEM) water electrolyzers, a feed of 0.1 M KOH is usually used (pH 13), or the local environment is shown to be lower pH ≈ 11 when deionized water is used instead of a concentrated alkaline feed.^20^ Operating with lower alkaline concentrations, such as 0.1 M, can provide benefits, including enhanced stability of the membrane and its functional groups.^21^ However, this lower pH environment can affect the oxygen evolution reaction (OER) by reducing the availability of OH^–^ ions on the catalyst surface, which can also affect restructuring, which may slow down the reaction and become a limiting factor in the process.^21,22^ Cerium oxide (CeO_2_) can act as a redox mediator and radical scavenger, providing desirable features such as rapid transformation between Ce^4+^ and Ce^3+^, which would benefit from a dynamic electron movement. Incorporating oxygen-deficient ceria with perovskite oxides significantly enhances the oxygen evolution reaction (OER) on a metal oxide- and hydroxide-based catalyst, with enhancement attributed to synergistic effects. Various reasons are given for the seen “synergistic effects”. The perovskite oxide has the main active sites, and ceria acts as a cocatalyst or support.^23,24^ The rich oxygen vacancies in ceria improve OER kinetics by increasing oxygen ion mobility and promoting beneficial interfacial charge transfer processes, which enhance electronic conductivity.^25^ The mixed electronic and ionic conductivity of CeO_2_ is rooted in the ability to release oxygen at higher temperatures or low oxygen partial pressure.^26^ Altering the oxidation state, e.g., Ni^3+^ to Ni^3±σ^, the associated Tafel slope of which was reported to be 81.9 mV/dec in Ni_*x*_Ce_*y*_ on carbon paper as an OER catalyst, exhibited high performance and durability in 1 M KOH.^27^ A change in the surface electronic structure of CeO_*x*_ leads to the possibility to tailor the Co^2+^/Co^3+^ redox, which reflects an increase in the oxygen vacancy content in prepared the hybrid nanostructure CeO_*x*_/CoS, achieving 10 mA/cm^2^ at an overpotential of 269 mV with a 50 mV/dec Tafel slope.^28^ During the oxygen evolution reaction, O^2–^/O^–^ species generated in defective CeO_2_ readily migrate from the CeO_2_ lattice onto the surface of RuO_2_.^29^ CeO_2_ acts as a redox mediator and an electron shuttle to supportively enhance the rate and reversibility of electron transfer in oxidation on poorly conducting metal oxides, peroxides, and superoxides.^30,31^ Recent studies further confirm that doping or modifying electrocatalysts with cerium oxide can significantly promote OER activity, primarily through the creation of oxygen vacancies and modulation of the electronic structure.^23,24,29,32–34^

In this work, we report the enhancement of the oxygen evolution catalytic activity of lanthanum manganese perovskite oxide (LaMnO_3_) in alkaline media by inclusion of various weights (up to 20%) of cerium oxide (CeO_2_). The codeposition or simultaneous synthesis of LaMnO_3_ (LM) and CeO_2_ was chosen over stepwise synthesis or post-mixing of the two oxides to enable homogeneous deposition of both oxides within proximity of each other at distances relevant to catalysis, therefore maximizing the possible synergistic effects present in the hybrid structure. We have shown that neither variation in oxygen vacancy concentration of the perovskite phase nor the oxidation state of the B-site transition metal of the as-prepared structure is directly linked to the enhancement observed. The optimized electrocatalyst containing ∼10 wt % of CeO_2_ content with lanthanum manganese perovskite (LCM-0.1) showed improved OER activity, achieving a greater than 22-fold increase in generated current density at 1.9 V versus RHE in 0.1 M KOH compared to pure LaMnO_3_. The observed enhancement in the electrocatalytic activity is attributed to the promotion of restructuring of the catalyst to form an OER active MnOOH structure, which is seen by in-operando Raman spectroscopy and ex-situ XPS measurements. Further, a water electrolyzer was fabricated using the optimized LCM-0.1 electrocatalyst, and the device performance was evaluated with different loadings of the LCM-0.1 catalyst in the electrolyzer anode. The results suggest that the incorporation or hybridization of cerium oxide in perovskite-based catalysts can be utilized as a method to promote restructuring to a more active MnOOH OER electrocatalyst.

## 2 Experimental Section

### 2.1 Materials

Lanthanum­(III) nitrate hexahydrate (99.90%), manganese­(II) nitrate tetrahydrate (98%), cerium­(II) nitrate tetrahydrate (99.5%), 20 wt % Pt/C, and 2-propanol were purchased from Alfa-Aesar, Thermo Fisher Scientific, U.K. Citric acid monohydrate (99.5%), mono ethylene glycol (99%), ethylene diamine (99%), hydrochloric acid (HCl, 35%), potassium iodide, sodium thiosulfate, Nafion-117 solution (Sigma-Aldrich, 5 wt % in H_2_O), and trimethylamine (40%) were purchased from Sigma-Aldrich.

### 2.2 Preparation of Lanthanum Manganese (LM) Perovskite with Ceria (CeO_2_)

The catalysts were prepared using sol–gel synthesis routes. Stoichiometric amounts of as-received lanthanum­(III) nitrate hexahydrate (99.90%, Alfa-Aesar), manganese­(II) nitrate tetrahydrate (98%, Alfa-Aesar), and cerium­(II) nitrate tetrahydrate (99.5%, Alfa-Aesar) were dissolved in an aqueous solution of citric acid monohydrate (99.5%) and mono ethylene glycol (99%) with a 3:1 molar ratio with respect to the total number of metal ions. The mixture was heated while stirring at 120 °C until the water evaporated, and a viscous gel was formed. The gel was further ignited to form an ash at 140 °C. Using a pestle and mortar, the ash was ground and transferred into a crucible for sintering at 900 °C for 12 h in air with a heating rate of 5 °C/min. The prepared powders were named based on the weight % of CeO_2_ loading in lanthanum manganese oxide (LM) perovskites, such as LM (LM-100 wt %), LCM-0.05 (LM 95 wt % and CeO_2_ 5 wt %), LCM-0.1 (LM 90 wt % and CeO_2_ 10 wt %), LCM-0.15­(LM 85 wt % and CeO_2_ 15 wt %), and LCM-0.2 (LM 80 wt % and CeO_2_ 20 wt %).

### 2.3 Characterization Techniques

X-ray diffraction analysis was used to investigate the crystal structure of the prepared electrocatalyst by using an X’Pert Pro diffractometer system (Panalytical) at room temperature using Cu-Kα radiation (λ_ave_ = 1.54 Å). High Score Plus software was used for the qualitative phase analysis.^35^ Quantitative analysis was carried out using Topas Academic (Version 6)^36^ and GSAS-II^37^ software. The Rietveld refinement^38^ technique was performed to determine the weight percentage of oxide phases, lattice parameters, and cell volume. The strategy involved setting the *R*3̅*cH* ^39^ space group for the lanthanum manganese oxide and using the *Fm*3̅*m* space group for CeO_2_.^40^ The refinement strategies involved scale factor, five-coefficient Chebyshev polynomial and 1/x background, a zero error, lattice parameters, and crystallite sizes. The physical information on powders such as surface area was obtained using Brunauer–Emmet–Teller (BET; Figures S1–S3). A Zetasizer instrument (ZTS1240- Malvern Panalytical, U.K.) was used to provide information on particle sizes (Figure S1b).

The iodometric titration method was used to determine the average bulk oxidation state of manganese in the perovskite. Concentrated hydrochloric acid was used to dissolve about 20 mg of powder under deaerated conditions. During the experiment, the Mn oxidation state was reduced to the lowest oxidation state (Mn^2+^), while the chloride ion was oxidized to Cl_2_ on the anodic side of the reaction (maintaining around 80 °C to minimize Cl_2_ dissolution in water and speed up the reaction). The produced chlorine gas was then transferred to a separate container with potassium iodide (0.1 M). At the final stage, sodium thiosulfate (0.01 M Na_2_S_2_O_3_) was utilized to titrate the iodine and starch solution, as the indicator was added close to the end point of titration. The oxidation state of manganese was then back-calculated using the amount of sodium thiosulfate used for the titration. See the Supporting Information for reaction details. In-situ Raman spectra were collected using a Horiba Yvon LabRam HR instrument with a 515 nm laser with 1.1 mW. For this experiment, the electrocatalysts were sprayed and coated on a titanium mesh, as was used for membrane electrolyte assembly (MEA) testing. The cell was assembled in the in-house-made electrolyzer with an active area of 1 cm^2^, and a quartz window was employed to allow laser access to the catalyst layer. Raman spectra were collected at various potentials of 0, 1.3, 1.6, and 1.9 V versus RHE. The objective lens was set at 100× magnification, and a 2400 g/mm grating was used. Scanning electron microscope (SEM) equipped with the energy dispersive x-ray spectroscopy (EDX) (Zeiss Sigma SEM with Gatan 3View, Gatan incorporation, Abingdon, UK) was used to evaluate the morphology of the electrocatalysts.

X-ray photoelectron spectroscopy (XPS) analysis of LM and LCM-0.1 before and after OER was performed by using a Thermo NEXSA, using monochromated Al Kα X-rays (1486.69 eV) at 6 mA emission. Source resolution for monochromated Al Kα X-rays is ∼0.3 eV and 12 kV HT, an elliptical spot size of 400 μm, and a 180° hemispherical analyzer in conjunction with a two-dimensional detector that integrates intensity across the entire angular distribution range. The instrument was calibrated to gold metal Au 4f (83.95 eV) and dispersion adjusted to give a BE of 932.6 eV for the Cu 2p_3/2_ line of metallic copper. The instrumental resolution was determined to be 0.29 at 10 eV pass energy by using the Fermi edge of the valence band for metallic silver.

### 2.4 Electrochemical Measurements

The electrochemical properties of the prepared catalysts were evaluated by using an electrochemical workstation with a Gamry potentiostat (Interface 5000E) instrument and an integrated frequency response analyzer (FRA). The ink composition consisted of 2 mg of the electrocatalyst, 0.1 mL of 2-propanol (Alfa-Aesar), 0.4 mL of deionized water (∼20 MΩ), and 60 μL of Nafion-117 solution (Sigma-Aldrich, 5 wt % in H_2_O) as the binder. 5 μL of the electrocatalyst ink was deposited on the gold electrode and dried in air. The gold electrode (3 mm diameter, mass loading fixed: 252 μg/cm^2^) was served as the working electrode, while a platinum sheet and Ag/AgCl leakless were used as the counter and reference electrodes, respectively. To activate the catalyst surface and ensure full oxidation of the OER catalyst, a chronoamperometry scan, holding potential at 1.85 V versus RHE for 15 min, was carried out before running the cyclic voltammetry (CV) measurements. During a CV run, voltage was swept from 0.4 to 1 V with reference to the Ag/AgCl electrode (corresponding to 1.4 and 2 V vs RHE) with scan rates of 5 and 1 mV/s, respectively. All of the potentials were converted and reported against the reversible hydrogen electrode (RHE). The environment of the electrochemical tests was 0.1 M KOH_(aq)_, which was purged with inert nitrogen gas for at least 20 min prior to the CV analysis. Electrochemical impedance spectroscopy (EIS) was used to evaluate the performance stability of the catalysts by comparing the impedance values in the low-frequency region as well as applying the *iR* correction on the data used for producing the Tafel slope. During the impedance measurement, the frequency ranging from 10^6^ to 10^–2^ Hz was used, and perturbation amplitudes of 10 mV and 1.85 V versus RHE were applied. The OER performance stability of the catalysts was studied with chronoamperometry at 1.85 V versus RHE for 60 min, during which changes in the delivered current density were monitored over time.

### 2.5 Fabrication of the Water Electrolyzer Device

The performance of electrocatalysts toward oxygen evolution was evaluated using a single cell made of titanium with a membrane electrolyte assembly (MEA). The area of the conductive part was 1 cm^2^ and coated with gold. KOH solution (1 M) was used as the electrolyte, and it was pumped to the anode and cathode separately with a serpentine flow pattern bordered by sealing the O-ring. The temperature of the cell (20, 40, and 60 °C) was controlled via thermostatic heat controllers inserted into the cell frame. Preparation of the cathodes involved a cathode loading of 0.4 mg_Pt_/cm^2^, and a carbon gas diffusion layer (GDL) with a microporous layer (MPL, nonwet proof, Freudenberg) was sprayed with a mixture of 20 wt % Pt/C and 28 wt % styrene–ethylene–butylene–styrene (SEBS) ionomer. The cathode was then immersed in a trimethylamine 25 wt % aqueous solution (Sigma-Aldrich) for about 72 h. As for the membrane, the radiation-grafted low-density polyethylene (LDPE)–vinyl benzyl chloride (VBC)–trimethylamine (TMA) was utilized as the alkaline anion exchange membrane (AAEM) with preparation details provided elsewhere.^20^ Anodes were prepared by spraying the synthesized electrocatalysts on a titanium fiber felt GDL (78% porosity, 0.3 mm thickness supplied by Bekaert Toko Metal Fiber Co., Ltd.). The prepared OER electrocatalysts were sprayed on titanium anodes to obtain loadings of 2.5 and 1.25 mg with 12.5 wt % SEBS ionomer. Identical to the cathode preparation, the anodes were also treated in TMA for about 24 h. All three components of the test were preactivated in a 0.1 M KOH solution for about 1 h before assembling the cell. The electrochemical testing of linear sweep voltammetry and impedance spectroscopy was carried out using an Autolab instrument (PGSTAT302 N).

## 3 Results and Discussion

### 3.1 Characterization and Structural Evaluation of the Electrocatalyst

Powder XRD patterns were obtained for the synthesized cerium lanthanum manganese oxides with different concentrations of the cerium nitrate precursor from 0 to 0.2 wt %, with results shown in Figure 1a.

**Figure 1:**
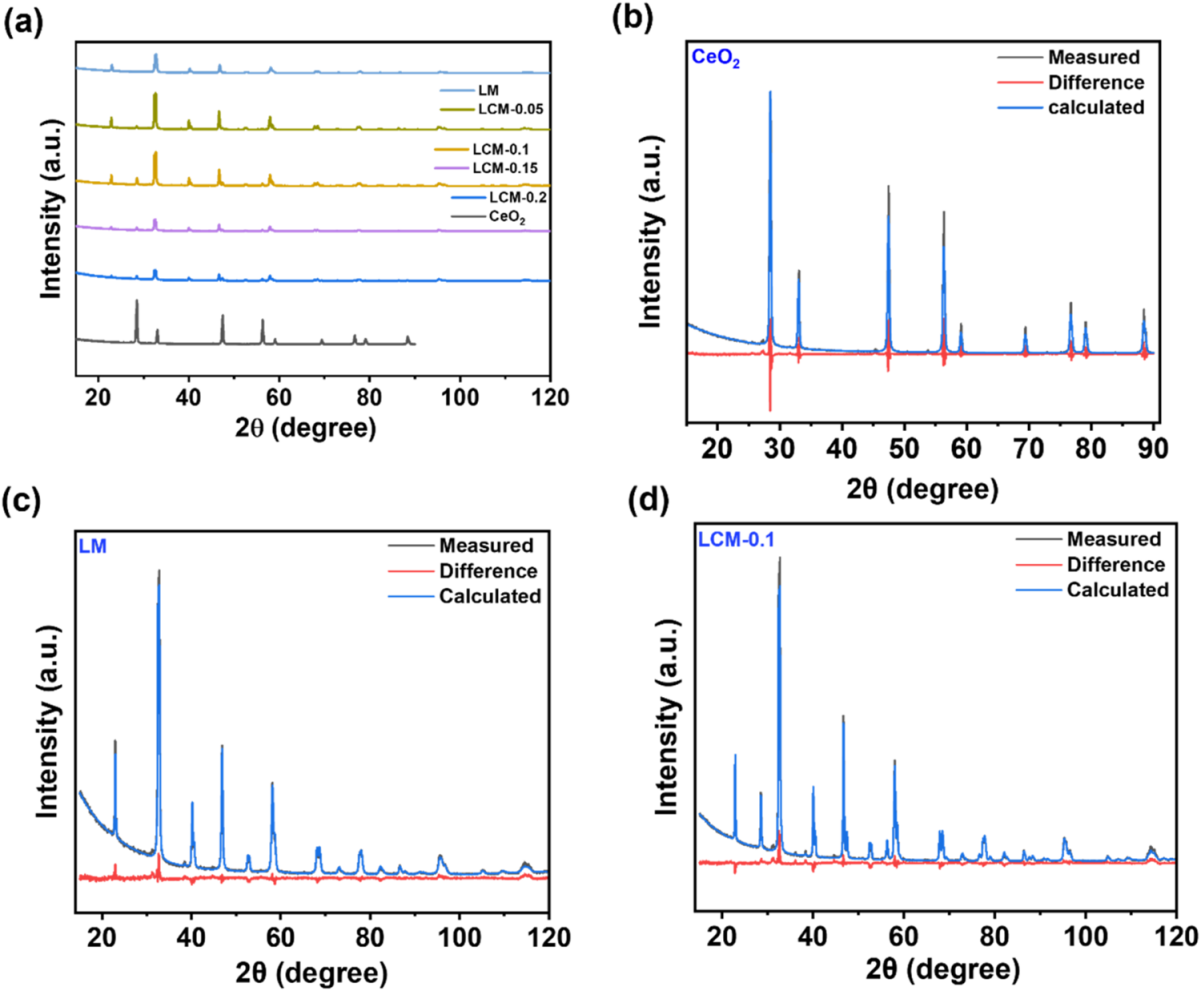
(a) X-ray diffraction pattern of lanthanum manganese-based perovskite oxides with cerium inclusion. Rietveld refinement graphs of (b) cerium oxide (100% of CeO_2_), (c) pure lanthanum manganese oxide (100% LM), and (d) lanthanum cerium manganese oxide with 10% intended cerium mixture (LCM-0.1:9.5% of CeO_2_ and 90.5% of LaMnO_3_).

The characteristic peaks of CeO_2_ (28.5° (111), 32.8° (200), 47.4° (220), 56.3° (311), 59.1° (222), 76.7° (331), and 79.0° (420)) and LaMnO_3_ (22.9° (100), 33.05° (110), 40.1° (111), 46.8° (200), and 58.1° (211)) are observed in Figure 1a. All of the reflections can be indexed based on the CeO_2_ centrosymmetric F-centered cubic structure with the *Fm*3̅*m* space group (ICSD No. 72155) and LaMnO_3_ structure (ICSD No. 258704) with the *R*3̅*cH* space group [(no. 167), (JCPDS file; 36-1092)].

To further understand the crystal structure and phase quantification, Rietveld refinement^38^ was carried out on the XRD patterns, and the results are summarized in Table 1 and Figures 1b–d and S6–S8. Figure S6 shows the Rietveld plot for the XRD data of LCM-0.05 collected at room temperature (weighted profile *R*-factor (*R*_wp_) = 8.08, and goodness-of-fit (GOF) = 3.37). The observed and calculated patterns are shown in blue and black lines, respectively. The calculated pattern is shown in blue, and the difference can be seen underneath (red). The vertical markers illustrate Bragg reflections. For Rietveld graphs of other samples, refer to Figures 1b–d and S6–S8 (Supporting Information). From Table 1, lanthanum manganese perovskite oxide (LM) was found to crystallize in the *R*3̅*cH* space group with *a* = 5.5143 ± 0.0002 Å and *b* = 13.3533 ± 0.0007 Å, which lie between the values of *a* = 5.525 Å and *c* = 13.353 Å (cell volume = 353.01 Å^3^) reported by Yang et al.,^41^ and *a* = 5.500 Å and *c* = 13.276 Å (cell volume = 347.90 Å^3^) reported by Huang et al.,^42^ for the same space group. Pure cerium oxide has shown a fluorite-type structure with lattice parameter *a* = 5.40855 Å, matching the value reported (*a* = 5.404 Å) by Zeng et al.^43^

**Table 1:** Rietveld Refinement Data Calculated on the X-ray Diffraction Patterns of Synthesized Lanthanum Manganese Oxides with and without Inclusion of CeO_2_ Content

sample	*a*_perovskite_ (Å)	*c*_perovskite_ (Å)	*V*_perovskite_ (Å^3^)	Gof	*R*_wp_ (%)	phase one (perovskite) (wt %)	phase two (cerium oxide) (wt %)	*a*_cerium oxide_ (Å)	*V*_cerium oxide_ (Å^3^)
LM	5.5143 ± 0.0002	13.3533 ± 0.0007	351.63 ± 0.03	2.0	6.4	∼100	0		
LCM-0.05	5.52270 ± 0.00013	13.3615 ± 0.0004	352.93 ± 0.02	2.9	8.4	95.8 ± 0.3	4.2 ± 0.3	5.4227 ± 0.0005	159.46 ± 0.04
LCM-0.1	5.52052 ± 0.00015	13.3653 ± 0.0004	352.75 ± 0.02	2.9	8.5	90.5 ± 0.3	9.5 ± 0.3	5.4190 ± 0.0002	159.135 ± 0.019
LCM-0.15	5.51979 ± 0.00015	13.3629 ± 0.0005	352.59 ± 0.02	1.7	5.8	87.2 ± 0.3	12.8 ± 0.3	5.4184 ± 0.0002	159.076 ± 0.019
LCM-0.2	5.51902 ± 0.00015	13.3610 ± 0.0005	352.45 ± 0.03	1.9	5.2	80.2 ± 0.3	19.8 ± 0.3	5.4181 ± 0.0002	159.051 ± 0.017
CeO_2_				4.3	11.5	0	∼100	5.40855 ± 0.00016	158.213 ± 0.014

As can be inferred from Table 1 and Figure S8, introducing cerium up to 20 atom % has resulted in a general increase in the lattice parameters and expansion of the perovskite cell compared to pure LaMnO_3_. Cerium oxide is detected as a separate phase. Quantification data show that the percentage of cerium oxide varies among the samples with an upward trend with increasing concentration of the cerium nitrate precursor (in the case of LCM-0.5, for example, 5 atom % cerium was the nominal percentage, which resulted in 4.20 wt % CeO_2_; details are provided in Table 1), suggesting that the majority of the used Ce has formed a separate CeO_2_ phase. An expansion in the unit cell parameter of the cerium phase can be noticed when compared to the pure cerium oxide. This might indicate that Ce^3+^ (ionic radius: 1.14 pm, Coordination No. (CN): 8) coexists in the crystal structure besides Ce^4+^ (ionic radius: 0.97 pm, Coordination No. (CN): 8). Ionic radius values were adapted from ref 44

Furthermore, a downward trend in the perovskite oxide’s lattice parameter “*a*” with increasing content of cerium oxide can be noted (see Figure S9a). The perovskite’s unit cell volume has shown a considerable increase once the cerium is introduced; however, it remains within a certain range with no apparent trend as the cerium oxide percentage increases. No detectable amount of Ce was inserted in the perovskite lattice. It should be noted that the cell expansion cannot be explained by^44^ Ce^3+^(ionic radius: 1.34 pm, Coordination No. (CN): 12)/Ce^4+^ (ionic radius: 1.14 pm, CN: 12) replacing La^3+^ (ionic radius: 1.36 pm, CN:12) sites in the lanthanum manganese perovskite lattice. The earlier observation regarding the perovskite oxide’s lattice parameter “a” might therefore be rooted in the nucleation, growth, and aging stages concerning the synthesis process, where the growth of metal oxides is generally diffusion-limited.^45^ Refined data (Table 1) show that samples contain different compositions of cerium oxide with about a 5 wt % interval.

Iodometric titration was carried out to provide compositional information on the perovskite phase (Table 2). The calculated average oxidation state of Mn was around 3+ with minimal diversion upon the change of the cerium oxide content. This does not account for oxygen vacancies, which are known to exist in LM structures.^46^ Moreover, the average value obtained does not provide any information on the presence of Mn^2+^ and Mn^4+^ species, which will be analyzed later by using XPS.

**Table 2:** Data from the Iodometric Titration Experiment Carried out on Pure Lanthanum Manganese Perovskite Oxide and Cerium-Containing Lanthanum Manganese Perovskite Oxides with Different Percentages

sample code	mg used	perovskite (mg)	perovskite (mol)	volume used (ml)	Mn oxidation state	standard deviation	Mn average oxidation state
LM	23.9	23.9	9.88 × 10^–05^	9.4	2.95	0.014	2.96
	22.1	22.1	9.13 × 10^–05^	8.7	2.95		
	25.5	25.5	0.000105	10.3	2.98		
LCM-0.05	25.2	24.0	9.93 × 10^–05^	10.3	3.03	0.03	3.01
	24.1	22.9	9.49 × 10^–05^	9.8	3.03		
	23.6	22.5	9.30 × 10^–05^	9.1	2.97		
LCM-0.1	23.5	21.0	8.70 × 10^–05^	9.1	3.04	0.023	3.02
	22.8	20.4	8.44 × 10^–05^	8.8	3.04		
	22.0	19.7	8.15 × 10^–05^	8.1	2.99		
LCM-0.15	23.8	20.1	8.33 × 10^–05^	8.2	2.98	0.044	3.03
	24.5	20.7	8.57 × 10^–05^	9.4	3.09		
	21.6	18.28	7.56 × 10^–05^	7.8	3.03		
LCM-0.2	25.5	20.7	8.57 × 10^–05^	9.2	3.08	0.004	3.08
	24.4	19.8	8.20 × 10^–05^	8.8	3.07		
	24.7	20.1	8.30 × 10^–05^	9	3.08		

Previous studies by Suntivich et al.^4,5^ suggested that the oxidation state of the B-site element of the perovskite oxide is the key determinant of the oxygen evolution catalytic activity. According to the iodometric titration data, the oxidation states of Mn and the perovskite’s oxygen vacancy concentrations of all the samples are very similar within the 5% experimental error associated with the titration experiment. This suggests that the samples might have a small amount of the Ce dopant or a small concentration of oxygen vacancies present. If a small amount of Ce^3+^ doping did take place below the detection level of 5%, this may result in a decrease in the Mn average oxidation state (from the presence of Mn^2+^); however, this cannot be detected by titration or XRD.

The substitution of Ce^4+^ at the A-site plays a crucial role in maintaining charge neutrality by minimizing the overoxidation of Mn to higher oxidation states and reducing the average Mn oxidation state in the lattice. As will be seen later in the XPS analysis, results reveal a reduction in the atomic % of Ce from 0.28 to below 0.1 (See Table 4) after the OER, indicating a minimal effect of Ce^3+^ doping. We also observed a significant change in the Ce^3+^/Ce^4+^ ratio, which decreases from 0.38 to 0.087 after the OER, while the Ce^4+^ content increases to 92% from 72.7%.

This would suggest that any differences in electrochemical properties that would be seen after accounting for differences in surface area should dominantly come from a change in intrinsic OER activity, which can be that of the as-prepared catalyst or restructured catalyst if restructuring was observed. Figures S1–S3 present the BET isotherm curves for the synthesized materials: LaMnO_3_ (LM), LCM-0.05, and LCM-0.1. The specific surface areas were determined to be 7.0350 m^2^/g for LM, 5.87 m^2^/g for LCM-0.05, and 6.46 m^2^/g for LCM-0.1. Notably, the addition of 10 wt % ceria in LCM-0.1 resulted in a surface area that remained comparable to that of pure LaMnO_3_, indicating that the incorporation of CeO_2_ did not significantly alter the overall surface characteristics. Furthermore, the average particle size distribution of LM was measured to be approximately 269 nm (Figure S1b), suggesting that the addition of CeO_2_ may influence the particle size without substantially affecting the surface area.

Figure S5a,b shows the SEM images of pure LaMnO_3_ and CeO_2_-incorporated LaMnO_3_, and the results reveal that pure LaMnO_3_ exhibits aggregated nanoparticles with irregularly shaped grains, characterized by a rough surface texture and well-defined grain boundaries. In contrast, CeO_2_-modified LaMnO_3_ (Figure S5b) displays a more compact and uniform particle arrangement, with refined and more homogeneous grain sizes compared to the pristine material. Elemental color mapping analysis confirms the uniform distribution of Ce within the LaMnO_3_ matrix, indicating the successful incorporation of CeO_2_ into the perovskite structure. These findings provide valuable insights into the surface area properties of the perovskite materials and their potential implications for electrocatalytic performance.

### 3.2 OER Performance of Electrocatalysts

#### 3.2.1 Effect of Cerium Oxide Incorporation

The OER activity of the prepared electrocatalysts was evaluated using cyclic voltammetry at a scan rate of 1 mV/s. The anodic sweep scan was presented with a 0.1 M KOH solution (Figure 2a,b and Table S1). The results indicate an enhancement in the OER activity with the inclusion of cerium oxide. There seems to be synergy that has led to a notable improvement in OER activity, as both oxides (LM and CeO_2_) individually have shown poor performance, as reported elsewhere.^9,34^ For example, at 1.8 V, current density increased from 0.005 to 0.141 mA cm^–2^ when using CeO_2_ (0.005 mA cm^–2^), pristine LM (LaMnO_3_, 0.0140 mA cm^–2^), and LCM-0.1 (0.141 mA cm^–2^), respectively. As shown in Figure 2b, OER activity at 1.8 V changed from 0.014 (LM) to 0.200 mA cm^–2^ (LCM-0.15), and at 1.9 V, the OER activity changed from 0.042 to 0.946 mA cm^–2^, showing the maximum activity at the 10% atomic ratio of CeO_2_ within the LCM-0.1 sample, beyond which no further improvement was seen or a decline in improvement was recorded.

**Figure 2:**
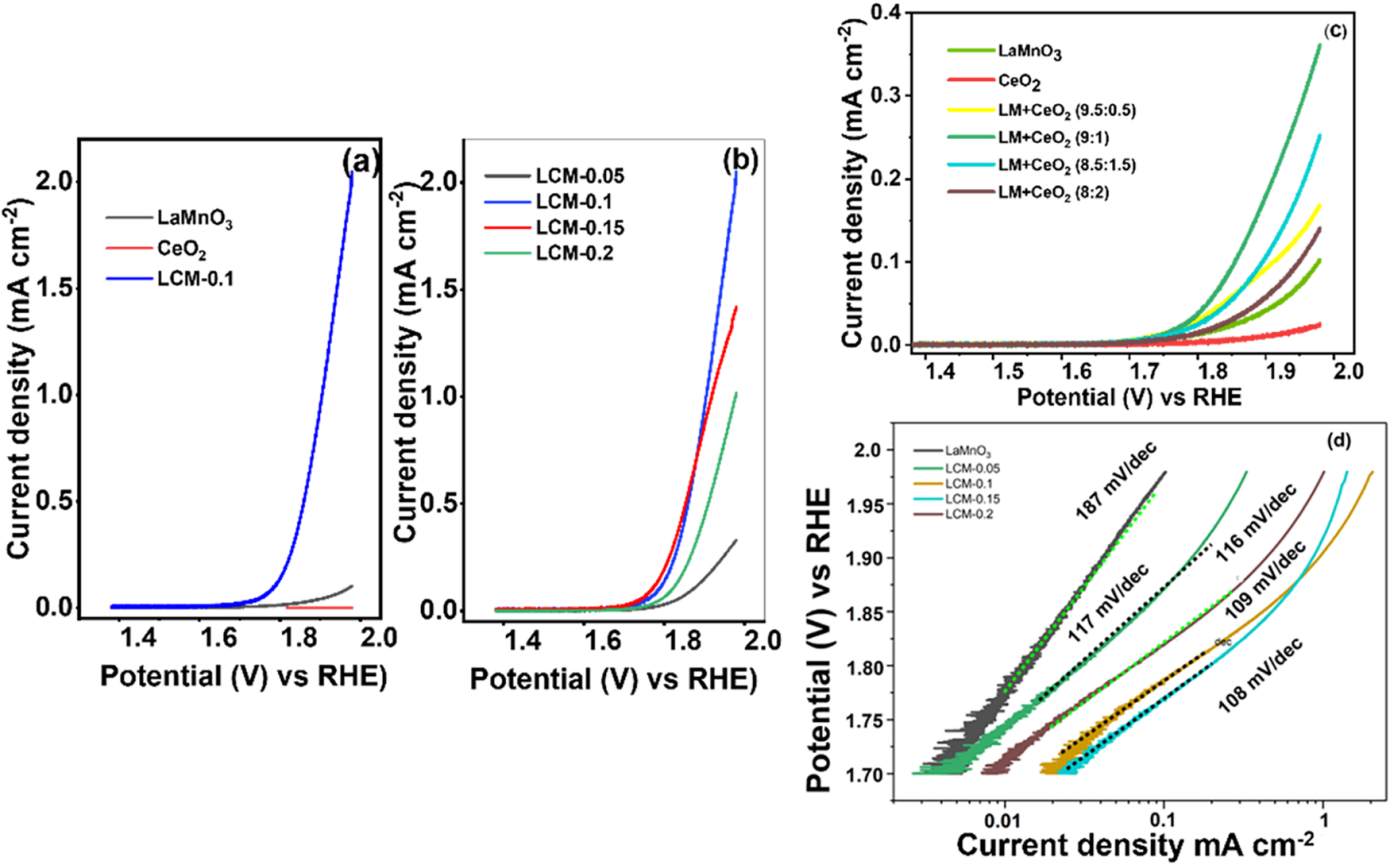
Oxygen evolution half-cell cyclic voltammetry (first cycle shown) measurement of (a, b) lanthanum manganese perovskite with inclusion of various weight % of cerium oxide, (c) physical mixture of lanthanum manganese perovskite oxide and cerium dioxide with different weight ratios in 0.1 M KOH alkaline media with a scan rate of 1 mV/s, and (d) Tafel plots obtained for lanthanum manganese perovskite with cerium oxide inclusion in 0.1 M KOH after IR correction.

The lack of OER activity on pristine CeO_2_ is anticipated due to the lack of unoccupied orbitals in their 3d energy layer that transition metals have, which is associated with their catalytic activity. Moreover, we measured the electrical conductivity of LM and CeO_2_. The low electronic conductivity of pure LM (7.49 × 10^–3^ S/cm) perovskite oxide can explain inadequate electrocatalytic performance (ceria 5.11 × 10^–8^ S/cm).

To understand if the seen enhancement was due to the low level of Ce doping below the detection level in the LCM-0.1 sample or due to some other synergistic effect from having two mixed phases of CeO_2_ and LM in close proximity,^25,29,42^ samples containing pristine LM (LaMnO_3_; 269 nm average particle size with a surface area of 7.035 m^2^/g as shown in Figure S1a,b) and CeO_2_ powder (305 nm average particle size) were physically mixed with various weight percentages to further explore the synergistic effects seen. Figure S9b shows the OER activity curves, where current density was normalized by BET surface area, result indicates, the LCM-0.1 sample demonstrated that improved intrinsic OER activity, achieved a current density of 0.000617 mA cm_BET_^–2^ at 1.8 V compared to LM (0.0000531 mA cm_BET_^–2^) and LCM-0.05 (0.000139 mA cm_BET_^–2^), and reaching 0.00704 mA cm_BET_^–2^at 1.95 V. Further, Table S2 (Supporting Information) provide the comparison of OER performance with recently reported perovskite catalysts. This indicates that the performance gain is not a surface area artifact but arises from enhanced intrinsic site activity and charge transfer kinetics attributable to improved electronic conductivity/redox mediation from CeO_2_ and facilitates lattice oxygen participation via oxygen vacancy formation.

Regarding calcination, 900 °C was chosen to ensure phase purity and structural robustness, as confirmed by XRD and Rietveld refinement. Lower calcination temperatures can lead to higher BET areas; however, they can also lead to incomplete perovskite phase formation and impurities/mixed phases, making it difficult to establish the reason behind the seen activity improvement or hindrance. Therefore, since the study focused on chemical structure and composition impact on activity, the calcination at 900 °C provided the most reliable phase-pure, stable catalysts, and the observed performance trends persisted even after BET normalization, underscoring that our improvements are intrinsic rather than driven by surface area differences.

Figure 2c shows the cyclic voltammetry results (forward scan rate: 1 mV/s) of the physical mixture of lanthanum manganese perovskite oxide and cerium oxide with different weight ratios. As can be inferred, the physical mixing of the oxides has also resulted in an improvement in OER electrocatalytic activity at 1.8 V versus RHE from 0.014 (LM) to 0.038 mA cm^–2^ (LM + CeO_2_: 9:1), although not as considerable as that seen in electrocatalysts prepared via the synthesis route (LCM-0.1; OER activity of 0.141 mA cm^–2^ at 1.8 V vs RHE). A similar trend was observed with the maximum enhancement of OER activity with the sample containing 10% CeO_2_. The larger improvement seen for codeposited cerium oxide and perovskite is expected due to the intimate contact and a higher number of contact sites between LaMnO_3_ and CeO_2_ oxides from homogeneous formation of both phases during the synthetic codeposition and sintering steps. The grain/phase boundary sizes are reduced, therefore improving the interface contact, the interaction between the two phases, and hence the OER activity.^47^ The decrease of the OER activity beyond 10% inclusion of CeO_2_ can be explained by the reduction of the volume fraction of Mn-based active sites and the loss of electronic conductivity in the mixture, outweighing any synergistic enhancement. Considering the enhancement seen in the OER activity from a physically mixed structure, this suggests that the reason behind the OER enhancement cannot be the cocatalysis of the OER, as suggested in some literature, as the physical distance between nanoparticles in a mixture is significantly higher than the bond length of O_2_. Moreover, any redox mediation will have a limited effect, since CeO_2_ is present as a solid sample with very limited contact with the LaMnO_3_ surface. This suggests that the reason behind the seen changes could be related to the so-called induction effect. For metal oxides, acidity and basicity are dependent on the charge and the radius of the metal ions, as well as the character of the metal oxygen bond. If the doping of the cation of one metal oxide to the structure of the other metal oxide during synthesis or if present within proximity where both were electrically connected, an induction effect between the two metal cations can occur, where the higher Lewis acidity of the metal substituent (e.g., Fe^3+^) compared with the parent metal (Ni^2+^) leads to the anodic shift (increase) of the Ni redox transition potential due to the inductive effect, as widely reported for Fe doping in NiOOH.^48,49^ Metal substituents with a higher electron affinity (stronger Lewis acids) than the parent metal can withdraw electrons from the parent metal cation, thereby reducing the energy of antibonding states. This leads to a decrease in the electron energy associated with the redox of M–ligand bonds and shifts the electrode potential in the positive direction. Herein, La and Ce (neutral) have similar electronegativity, which is lower than that of manganese. This means La^2+^ and Ce^3+^ also have lower negativity than Mn^3+^. However, Ce^3+^ has a higher electronegativity than La^2+^ (due to a higher oxidation state). This means it is expected that samples containing La- and Mn-based oxides are expected to shift Mn redox potential (2 to 3 and 3 to 4) to lower potentials than those of Ce–Mn oxide. Having some of Mn (B-site) in a lower oxidation state, i.e., 2+ (which can be accompanied by oxygen vacancies), or retaining a lower oxidation state of 3+ at higher potentials was suggested to play an important role in OER activity, as discussed earlier in the literature,^4,5^ and could lead to improved OER activity. It is interesting to note that the seen enhancement in the OER is accompanied by an enhancement in the apparent Tafel slope (Figure 2d) between LaMnO_3_ samples and those containing CeO_2_, e.g., LCM-0.1. This suggests that the rate-limiting step or surface participating in OER is different between the two samples and that the increase in activity is not due to the increase in activity (exchange current density) or surface area alone.

Another explanation is that the presence of CeO_2_ could influence or promote the restructuring of the prepared catalyst under oxidative conditions (potentials) where the OER takes place. For example, we have shown that the α-MnO_2_-based electrocatalyst restructures during OER with notable changes to the crystal structure in 0.1 M KOH with a significant increase in 300 and 310 facet contents, suggesting dissolution of Mn ions in solution and redeposition on the catalyst surface with preferable orientation, accompanied by an increase in the Mn average oxidation state and a decrease in electronic conductivity.^19^ The restructuring of the electrocatalysts will be discussed later in the in-situ Raman analysis and XPS spectroscopy Sections 3.2.3 and 3.2.4.

The evolution of oxygen occurs through a multi-electron, complex electrochemical reaction, where different pathways and rate-determining steps (RDSs) have been proposed based on the electrocatalyst type, electrolyte, and the range of applied overpotential.^14,50–52^ In alkaline media, the overall reaction can be written as $2{\mathrm{OH}}^{-}\to H_{2}O+\frac{1}{2}O_{2}+2e^{-}$.

Data from Tafel extrapolation (IR-corrected) in Table S1 show a Tafel slope of 187 mV/dec for LaMnO_3_, which is closer to 154 mV/dec reported by Yamada et al.^53^ compared to 334 mV/dec by Liu et al.^13^ The Tafel slope values higher than 120 mV/dec could be caused by poor electronic conductivity and an additional process taking place for example oxidative restructuring or the change in the Mn oxidation state, resulting in the change of the apparent slope of log current density–potential. Upon incorporation of cerium oxide, the Tafel slopes decreased by 37, 45, 46, and 38 mV/dec for LCM-0.5, LCM-0.1, LCM-0.15, and LCM-0.2, respectively. The observed range (∼120 mV/dec) for the Tafel slope might indicate that the first electron transfer AS + OH^–^ → ASOH + *e* ^–^ is the rate-determining step (RDS), as suggested elsewhere.^54^ However, a high Tafel slope could be caused by the high coverage of intermediates, e.g., the Tafel slope for the third electron transfer step is ca. 30 mV/dec at a low surface coverage of intermediates, θ < 0.3. This increases to 120 mV/dec when θ > 0.3.^21^ As discussed above, the Tafel slope is also influenced by other processes than the OER (adsorption or oxidation), limiting the extent to which it can be used to discuss the mechanism and rate-limiting step. It is, however, interesting to see that the improvement in the Tafel slope on ceria addition was only seen in the potential range up to 1.85 V, above which the Tafel slope became similar to that of LM, while the exchange current density remained higher (Figure 2d). The higher exchange current density could be the effect of mediation on the OER. The change in Tafel slope could be linked with the effect of increased potential on the Ce oxidation state (Ce^3+^ ↔ Ce^4+^ + *e* ^–^ *E*_0_ = 1.72 V vs RHE) and/or that of the B-site, where higher potentials force higher Ce^4+^ and Mn^4+^, limiting mediation and the OER prospects.

#### 3.2.2 Electrocatalyst Stability and the Effect of Temperature

Figure 3 shows the impedance spectroscopy and chronoamperometry of the prepared perovskite oxide with different wt % of CeO_2_. Corresponding charge transfer resistance, *R*_p_ values reported here at a potential of 1.85 V versus RHE from the three sequences of impedance spectroscopy measurement, and data obtained from the equivalent circuit fit using the simple Randles circuit model are shown in Figure S15. Values of *R*_p_ along with double-layer capacitance *C*_dl_ and ionic resistance *R*_s_ are listed in Table 3. Values of *C*_dl_ and *R*_s_ were similar, as expected, between the studied samples. However, *R*_p_ values were different and followed the expected trend of sample LCM-0.1, followed closely by LCM-0.15, having lower charge transfer resistance among the studied samples, with charge transfer resistance 24 times lower than that of the LMnO_3_ sample (0.12 vs 2.94 ohm cm^2^). This is consistent with the 22-fold increase in current density reported earlier. The initial measurement (*R*_p‑initial_) was conducted after 15 min of chronoamperometry at 1.85 V but before subjecting the sample to potential cycling. Two aging protocols are adopted first, involving cycling the electrode between 1.23 and 2.0 V versus RHE at a scan rate of 5 mV/s for 5 full cycles and collecting the impedance spectra and the secondary aging protocol, where the electrode is held afterward at 1.85 V versus RHE for 1 h (Figure 3b). The reported changes in charge transfer resistance are *R*_p‑cycling_ and *R*_p‑chronoamperometry(CA)_, respectively.

**Figure 3:**
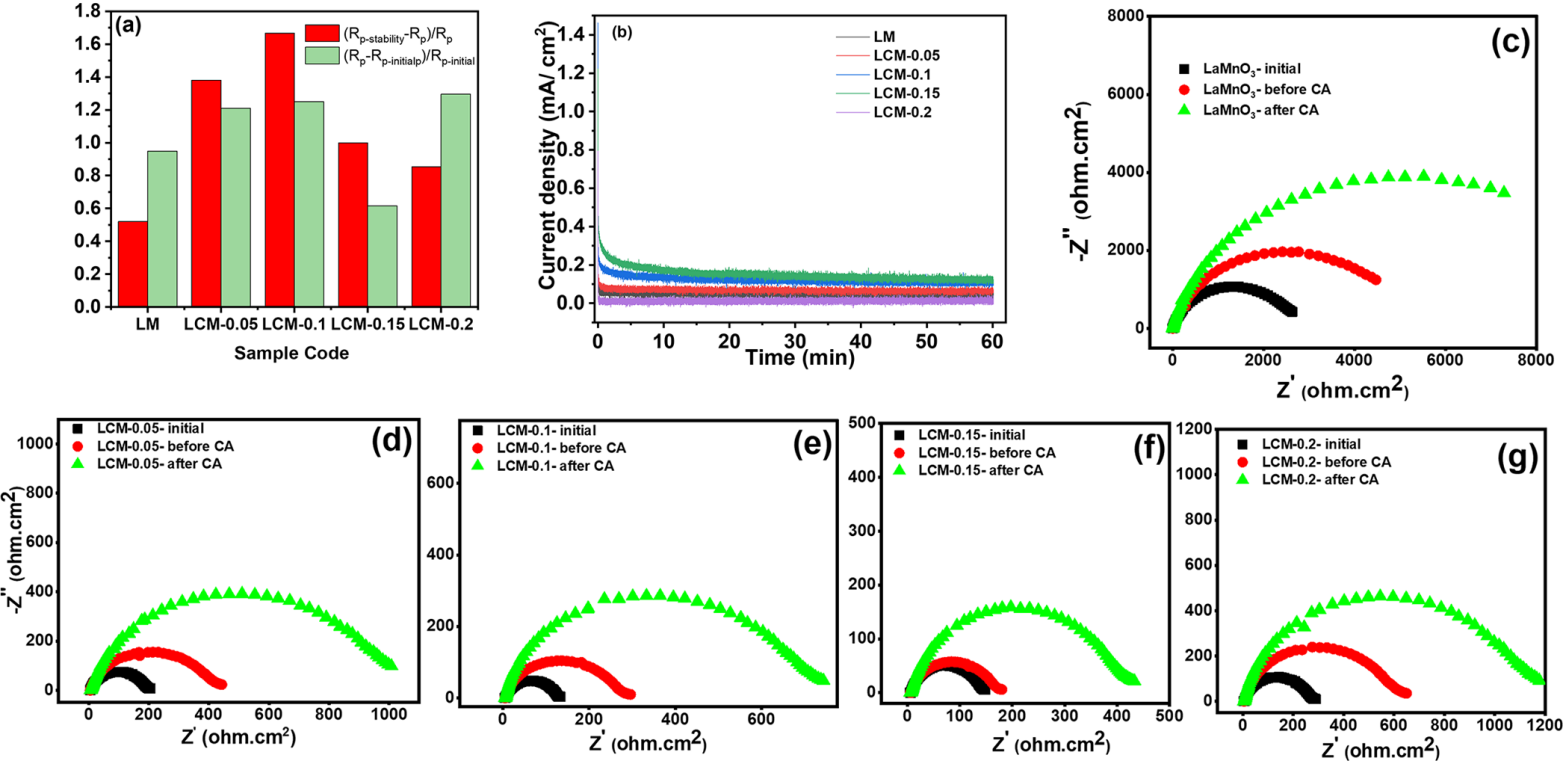
Electrochemical data collected on the half-cell study of electrocatalysts in 0.1 M KOH at room temperature. (a) Early stage stability indicator plot where green bars show first phase stability and red bars show stability after chronoamperometry (CA) for 1 h at 1.85 V. (b) CA scans at 1.85 V for 1 h. Nyquist plot from impedance spectroscopy measurement for (c) LaMnO_3_ at three stages of initial, before CA, and after CA. (d–g) Nyquist plot from impedance spectroscopy measurement at three stages of initial, before CA, and after CA for LM, LCM-0.05, LCM-0.1, LCM-0.15, and LCM-0.2, respectively.

**Table 3:** Table of Electrochemical Data Obtained from Impedance Spectroscopy and Corresponding Equivalent Circuit Fit

sample code	*C*_dl_ (F/cm^2^) initial	*C*_dl_ (F/cm^2^)	*C*_dl_ (F/cm^2^) after CA stability	*R*_p_ initial (kohm·cm^2^)	*R*_p_ cycling (kohm·cm^2^)	*R*_p_ after CA stability (kohm·cm^2^)	*R*_s_ (ohm·cm^2^)
LM	8.61 × 10^–05^	9.48 × 10^–05^	9.73 × 10^–05^	2.94	5.73	8.71	4.94
LCM-0.05	1.03 × 10^–04^	1.05 × 10^–04^	0.000112	0.19	0.42	1.00	5.45
LCM-0.1	7.18 × 10^–05^	9.02 × 10^–05^	8.8 × 10^–05^	0.12	0.27	0.72	5.44
LCM-0.15	1.14 × 10^–04^	1.16 × 10^–04^	0.000105	0.13	0.21	0.42	5.99
LCM-0.2	6.50 × 10^–05^	7.14 × 10^–05^	7.69 × 10^–05^	0.27	0.62	1.15	4.87

As can be inferred from Table 3 and the impedance spectra in Figure 3a,c–g, significant changes (increase) to the value of charge transfer resistance can be seen with aging, with values doubling from their original value after the 5 cycle protocol and doubling again after 1 h potential hold at 1.85 V. Figure S9c illustrates the stability evaluation of LM and LCM-0.1, conducted via chronoamperometry at 1.85 V versus RHE for a duration of 11 h in 0.1 M KOH. For LCM-0.1, the initial current density of 0.0814 mA cm^–2^ remained essentially unchanged during the first 1 h of operation. Over extended testing (11 h), the current density declined to 0.047 mA cm^–2^, representing a reduction of approximately 42% relative to the starting value. This raises questions on the stability of MnOOH species and MnO_2_ generally at pH 13, highlighting that transition metal oxides with good stability and activity in an alkaline water electrolyzer (7 M) might not be adequate for use in AEM electrolyzers running at pH 13. During the sweep to positive potentials from rest potentials to drive OER oxidation within the structure of materials is anticipated Mn^2+^ to Mn^3+^ and Mn^3+^ to Mn^4+^ and Mn^4+^ to Mn^6+^ and Mn^7+^ (above 1.6 V RHE) and Ce^3+^ to Ce^4+^ within studied potential range of 1.23–1.9 V RHE.^38^ This would mean that the composition of the material and the original average oxidation state of elements with the potential redox couple might change during the aging and testing protocols as well as the potential loss into the solution (dissolution) of Mn^6+^/Mn^7+^. Mn^4+^and Mn^3+^ on the other hand are stable as oxides in pH 13–14 tested.

It is interesting to note that the ratio of charge transfer resistance increases (degradation) in the CeO_2_-free sample, i.e., LaMnO_3_. After the first and second aging steps, the values were 1.95 and 1.52, respectively, similar to those for the LCM-0.1 sample (2.2 and 2.36, respectively). The sample with a CeO_2_ content of ∼15 wt % showed loss ratios of 1.61 and 2, respectively, but the lowest charge transfer resistance after two aging protocols, which is in agreement with cyclic voltammetry graphs in Figure 2b.

#### 3.2.3 In-Situ Raman Analysis

To provide insights into the possible catalyst restructuring processes and in an attempt to understand the observed enhancement in the OER with CeO_2_ addition into LM, Figure 4a,b shows the in-situ Raman spectra of LM and LCM-0.1. The samples were coated on a titanium mesh and tested in 0.1 M KOH.^55^ The electrode potential was increased with a 300 mV interval starting from 1.3 to 1.9 V, and Raman spectra were collected. Figures S10 and S11 show frame-by-frame spectra capturing the evolved oxygen during the in-situ Raman spectroscopy experiment. Starting with the CeO_2_-free sample of LaMnO_3_ (Figure 4a), the Raman spectra is consistent with reported LaMnO_3_.^56^ The peak at ∼246 cm^–1^ is attributed to native titanium oxide covering the titanium fiber substrate at which the catalyst was deposited.^57^ Further, LaMnO_3_ in an alkaline medium can lead to the formation of La­(OH)_3_, which exhibits Raman active modes at approximately 273, 334, and 456  cm^–1^, corresponding to the A_1g_, E_2g_, and E_1g_ vibrational modes, respectively.^58,59^ However, if La­(OH)_3_ forms on the catalyst surface, it typically produces only weak or broad Raman signals, which are often overshadowed by much stronger Mn–O vibrational bands. Additionally, the peak at 246 cm^–1^ was visible prior to increasing potential.

**Figure 4:**
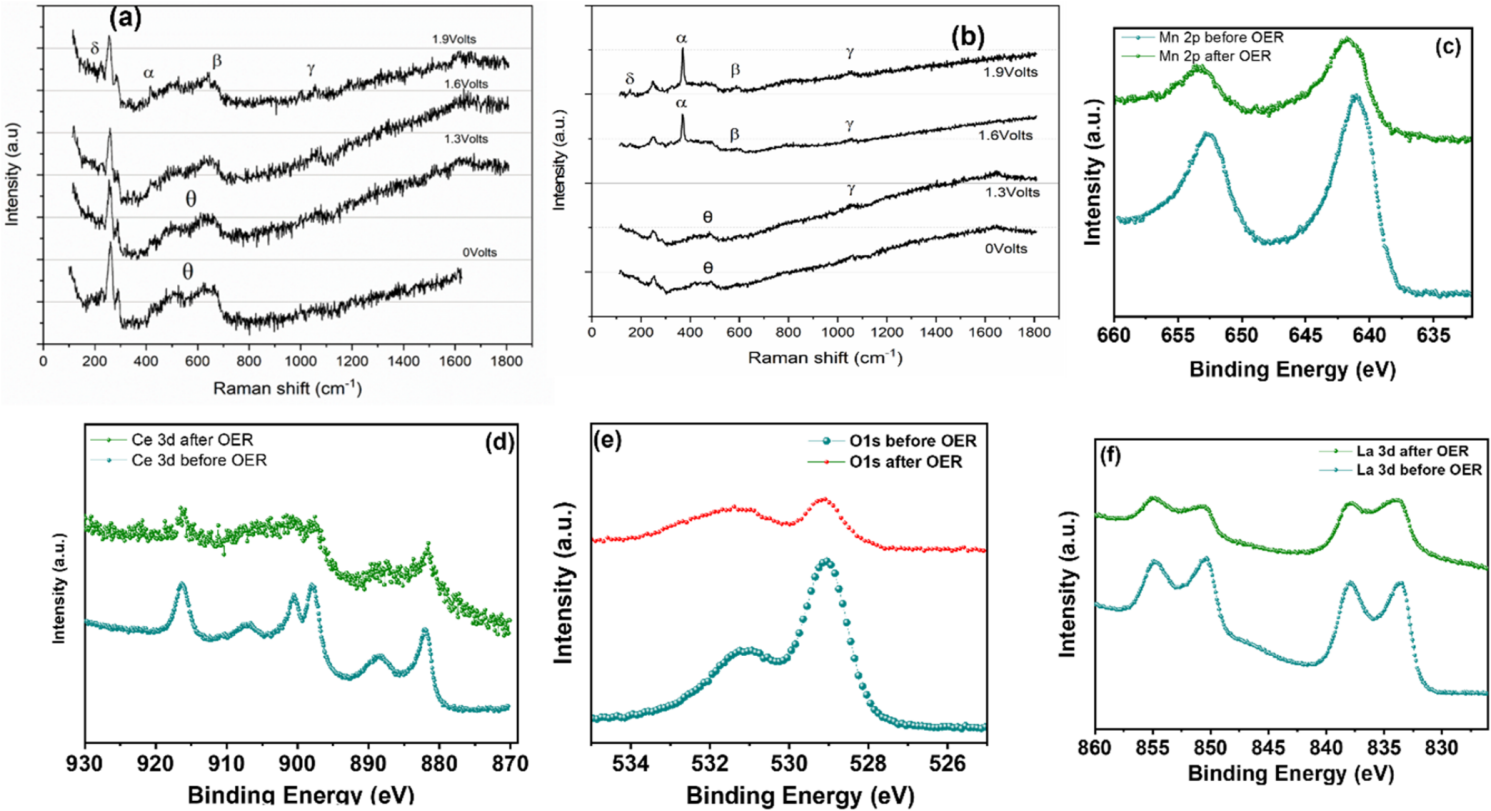
(a) In-situ Raman spectroscopy of (a) LM and (b) LCM-0.1 electrocatalysts coated on a titanium mesh at various voltages in 0.1 M KOH. The XPS spectra of the (c) Mn 2p, (d) Ce 3d, (e) O 1s, and (f) La 3d peaks of LCM-0.1 before and after the OER test.

The two broad peaks in the 400–700 cm^–1^ region of the spectrum (labeled in Figure 4a as θ) characterized by at 538 and 665 cm^–1^ are attributed to the E_g_ bands defined by the vibration of oxygen in MnO_6_ octahedra stretching mode in both rhombohedral phase (*R*3*c*) LaMnO_3_ ^56,60^ and other Mn oxide structures such as tetragonal structure, (*P*42/*mnm* space group), orthorhombic structure (*Pbnm* space group), or ramsdellite defective structure representing β-MnO_2_, R-MnO_2_, and γ-MnO_2_, respectively.^61^ The peaks in the 400–700 cm^–1^ region remain visible upon an increase in potential to 1.6 and 1.9 V versus RHE, with very small new peaks appearing at ca. 1065 cm^–1^ (labeled γ in Figure 4a), which is usually associated with OH, as will be discussed further below.

On the other hand, sample LCM-0.1 had weaker peaks in the 400–700 cm^–1^ region of the spectrum (labeled in Figure 4b as θ), and at 1.6 and 1.9 V versus RHE, a very distinctive strong peak at 365 cm^–1^ belonging to groutite (α-MnOOH) and the Bg mode assignment^62^ (labeled α in Figure 4b) in addition to a weaker peak at 1065 cm^–1^ (labeled γ in Figure 4b) can be seen. The very strong peak at 385 cm^–1^ is a unique feature, suggesting formation of α-MnOOH (groutite) species, which are known to be formed from Mn^2+^ oxidation in mild alkaline conditions.^63–65^

Manganite (γ-MnOOH) is isostructural with pyrolusite, and groutite (α-MnOOH) with ramsdellite, but in each case with Mn^3+^ instead of Mn^4+^, and OH^–^ replacing half of the O atoms. Groutite has the same symmetry as ramsdellite (*Pnma*) but has six more Raman active modes due to the additional OH. The groutite spectra collected under parallel polarization conditions reveal that the Mn–O stretch modes between 500 and 650 cm^–1^ are A_g_ modes (symmetric), and those at 97, 385, and 485 cm^–1^ are B_g_ modes (asymmetric) with the mode at 1065 cm^–1^ representing OH bending with motion in the tunnel direction.^62^ In other resources, the peak at 1057 cm^–1^ was assigned to a superoxide MnOO^–^ species similar to the cobalt superoxide species of CoOO^–^ reported at 1075 cm^–1^.^66^ The peaks at 368 and 587 cm^–1^ can be associated with Mn^3+^(O)­(OH), which have also been observed by others.^67,68^

The addition of CeO_2_ therefore seems to promote MnOOH formation at oxidative conditions (higher potential and O_2_ presence) in alkaline media. The formed MnOOH is known to be an active OER catalyst, which explains the improvement in the performance of the OER over that of LaMnO_3_. It is not clear if the addition of CeO_2_ lowers the oxidation potential from Mn^2+^ to Mn^3+^ through the inductive effect or if the seen effect comes from a small amount of doping of Ce^3+^ in the A-site, resulting in the presence of higher content of Mn^2+^ in the LCM-0.1 sample, or if Ce^3+^/Ce^4+^ redox promotes restructuring (dissolution and redeposition) of Mn active species. However, the inductive effect does not explain the observed OER enhancement for the physical mix of CeO_2_ and the LaMnO_3_ sample. Some proposed mechanisms for oxygen evolution in alkaline media suggest that the rate of the reaction depends on the extent of adsorption of OH^–^ on the active site, as well as the pace of electron transfer between the reaction intermediates. The concentration of OH^–^ affects the performance of the OER catalyst by influencing the deprotonation of OOH. In the higher pH electrolyte, NiOOH is more likely to deprotonate and change to NiOO^–^, which is a precursor of O_2_ production.^69^ The same mechanism is also suggested for MnO_2_ with an OER Tafel slope of 30 mV/dec at a low current density, increasing to 60 mV/dec at a high current density in 0.1 M KOH.^19^ It is further proposed that the higher content of Mn^3+^ in MnO_2_ than that in Mn^4+^ will result in higher defects, improving the catalytic performance at the cost of electronic conductivity.^70^ However, this was not seen elsewhere.^19^ Moreover, the enhancement in the OER activity was seen in both 0.1 and 1 M alkaline solutions, where high OH^–^ surface coverage on all samples is expected. From the Raman spectroscopy results, we postulate that MnOOH is the active OER species and that the OER mechanism aligns with the mechanism proposed by Kobussen^71^ (eqs 1 to 5) as the dominant/occurring OER pathway in contrast to that of pristine lanthanum manganese perovskite that did not undergo restructuring which is reported to undergo LOER mechanism.

1 $$\mathrm{AS}+{\mathrm{OH}}^{-}\to \mathrm{ASOH}+e^{-}$$

2 $$\mathrm{ASOH}+{\mathrm{OH}}^{-}\to \mathrm{ASO}+H_{2}O+e^{-}$$

3 $$\mathrm{ASO}+{\mathrm{OH}}^{-}\to \mathrm{ASOOH}+e^{-}$$

4 $$\mathrm{ASOOH}+{\mathrm{OH}}^{-}\to {\mathrm{ASOO}}^{-}+H_{2}O$$

5 $${\mathrm{ASOO}}^{-}\to \mathrm{AS}+O_{2}+e^{-}$$

­(AS: active site)

The perovskite surface restructuring could be an independent process involving the oxidation of the catalyst, dissolution, and redeposition as Mn oxyhydroxide or perovskite.^72,73^ Therefore, we expect to see two dynamic processes in terms of the time scale of testing. In a short window of a few cycles/minutes, depending on the hydroxyl concentration or the range of applied potential, the perovskite structure undergoes amorphization, leading to the formation of a hydrous amorphous layer containing manganese cations. The nature of the layer would be a factor of the perovskite structure underneath/the structure from which the layer is formed. The metal cation (Mn) of this layer is believed to undergo redox reactions (for example, Mn^3+^ ↔ Mn^4+^ + *e* ^–^), catalyzing or mediating the oxygen evolution process. The other process is related to the dissolution of the metal cation and redeposition, which can also affect the bulk of the catalyst. This will be discussed further below.

#### 3.2.4 Surface X-ray Photoelectron Spectroscopy before and after OER

Before and after the OER, the chemical states of LM and LCM-0.1 were investigated through surface X-ray photoelectron spectroscopy (XPS) analysis. The analysis was performed before and after the OER, which involved 10 cycles of cyclic voltammetry (CV) and chronoamperometry at 1.85 V in a 0.1 M KOH solution for 30 min. The results are presented in Figures 4c–f and S12–S14.

Figures S12 and S13 and Table 4 show the XPS survey spectra of LM and LCM-0.1 and the changes in the elemental composition before and after the OER. In LM, the deconvoluted Mn 2p spectra reveal that the Mn^3+^ fraction decreases from 48.9% to 33.2%, whereas Mn^4+^ increases from 51.1% to 66.8% after the OER (see Figure S12b and Table 5). Additionally, the Mn 2p peak shifts toward higher binding energies, indicating the oxidation of Mn^3+^ to Mn^4+^. Furthermore, the Mn 2p peaks become more asymmetric and show reduced intensity, attributed to the dominant presence of the Mn^4+^ species. The atomic percentage of Mn shows a significant decrease from 0.46 to 0.055 [normalized by atomic % of La] after the OER, while the La content simultaneously increases from 11.9% to 14.6%, as shown in Table 4. The loss of Mn from the surface is likely due to oxidation of Mn^4+^ to Mn^6+^ and Mn^7+^ soluble species, resulting in La enrichment and the concurrent formation of La­(OH)_3_, as supported by the literature^74^

**Table 4:** XPS Survey Spectrum Details before and after the OER of LM and LCM-0.1

samples code	atomic % of La	normalized by atomic % of La			
		La	Mn	O	Ce
LM before OER	11.9	1.0	0.46	6.90	
LM after OER	14.6	1.0	0.055	5.78	
LCM-0.1 before OER	7.0	1.0	1.1	10.75	0.28
LCM-0.1 after OER	13.6	1.0	0.67	5.96	below 0.1

**Table 5:** XPS Mn 2p and O 1s Peak Details before and after the OER of LM

	atomic % of element in LCM					
	Mn 2p in %			O 1s peaks in %		
samples details	Mn^3+^	Mn^4+^	Mn^3+^/Mn^4+^	M–O	M–OH	M-H_2_O
LM before OER	48.9	51.1	0.9569	29.8	69.3	0.9
LM after OER	33.2	66.8	0.4970		76.9	23.1

Figure S12d illustrates the deconvoluted La 3d spectra before and after the OER. The XPS characteristic peaks of La 3d_5/2_ (∼834–838 eV) and 3d_3/2_ (∼850–855 eV) show reduced intensity and peak broadening, indicating surface transformation, i.e., restructuring upon Mn loss and formation of La­(OH)_3_, resulting in an increase in La content (see Table 4).^74,75^ Since La­(OH)_3_ is insoluble in alkaline media, it remains on the catalyst surface as a stable hydroxide layer, resulting in a decrease in the main La 3d peak intensity.^75,76^ Additionally, the O 1s spectra (see Figure S12e) show that the peak at ∼528 eV, associated with lattice oxygen (M–O bonds), disappears after the OER. In contrast, the peak corresponding to metal hydroxide (M–OH) species significantly increases, from 69.3% to 76.9%, as shown in Figure S12e. This shift supports the formation of surface metal hydroxides, including La­(OH)_3_, indicating substantial surface transformation during the OER.^74^

The deconvoluted XPS Mn 2p_3/2_ spectra of LCM-0.1, both before and after the OER, are presented in Figure S14a–b. The deconvoluted peaks at ∼640–642 eV are attributed to Mn^3+^, ∼638–639 eV belong to Mn^2+^, and ∼643–644 eV belong to Mn^4+^ in LaMnO_3_. These results confirm the Mn oxidation states as Mn^3+^ (∼75%), Mn^2+^ (∼9%), and Mn^4+^ (∼16%),^77,78^ which is consistent with the average oxidation state results of iodometric titration data (see Table 6). This shows the importance of XPS in deconvoluting different Mn oxidation states present. The presence of 9% Mn^2+^ is significant, as this can transform to MnOOH on cycling. This highlights the importance of characterizing surface species and their oxidation state, as they will play a key role in restructuring and surface transformation, which will affect catalytic activity significantly. It is evident that relying on bulk characterization alone and the average oxidation state will miss important information, e.g., the presence of Mn^2+^ on the surface or the change of the atom % of Mn, Ce, and La on the surface after restructuring, which are important evidence of ongoing surface restructuring.

**Table 6:** XPS Mn 2p, O 1s, and Ce 3d Peak Details before and after the OER of LCM-0.1

	deconvoluted XPS peaks in LCM–0.1(%)									
	deconvoluted Mn 2p peaks				deconvoluted O 1s peaks			deconvoluted Ce 3d peaks		
samples conditions	Mn^2+^ (%)	Mn^3+^ (%)	Mn^4+^ (%)	Mn^3+^/Mn^4+^	M–O (%)	M–OH (%)	M-H_2_O(%)	Ce^4+^ (%)	Ce^3+^ (%)	Ce^3+^/Ce^4+^
before OER	9	75	16	4.68	55	39.5	5.5	72.7	27.3	0.38
after OER	-	56.6	43.4	1.304	38.8	48.2	13.0	92	8	0.087
references	^77,78^	^77,82,78^	^77,78^	^77,78^	^82^	^82^		^83^	^83^	^83^

Following cyclic voltammetry (CV) and chronoamperometry at 1.85 V in a 0.1 M KOH solution for 30 min, the catalyst surface undergoes partial hydroxylation, i.e., formation of surface-bonded–OOH/–OH species. Simultaneously, lattice oxygen is partially consumed (oxidized) and replaced by −OH groups on the surface with three distinct oxygen XPS peaks of (M: metal) M–O, M–OH, and H_2_O. After the OER, the Mn 2p_1/2_ and Mn 2p_3/2_ peaks shift toward higher binding energies, with a slight reduction in intensity and peak broadening width compared to those before the OER. These XPS features reflect that the LCM-0.1 surface becomes richer in Mn when the Mn concentration on the surface decreased slightly from 1.1 to 0.67 [normalized by atomic % of La]. (See Table 4).

The post-OER deconvoluted XPS Mn 2p_3/2_ spectrum of LCM-0.1 confirms that the Mn oxidation state increases, with Mn^4+^ rising to 43.4% from 16%. In MnOOH, Mn is initially in the +3 oxidation state and can be oxidized to Mn^4+^ when a higher potential of 1.85 V versus RHE is applied.^78,79^ The formation of hydroxyl and oxy hydroxyl species and the presence of MnOOH on the catalyst surface are further confirmed by in-situ Raman spectroscopy (see detailed discussion in the Section 3.2.3).

Figure S14g shows the deconvoluted spectra of Ce 3d. Deconvoluted 3d_5/2_ peaks confirm the peaks at ∼881.5 eV, characteristic of Ce^3+^, and the peaks at ∼883.0 and 887.9 eV belong to Ce^4+^ in LaMnO_3_ with 10% CeO_2_.^80,81^ After the OER, the XPS Ce 3d spectrum (Figure S14h) moved to higher binding energy with reduced overall intensity, indicating oxidation of Ce^3+^ to Ce^4+^ with a notable shift in the Ce^3+^/Ce^4+^ ratio from 0.38 to 0.087, while Ce^4+^ increases from 72.2% to 92% (see Table 6). The decrease in surface Ce atomic concentration, given the low solubility of CeO_2_ in pH 13, must be due to reconstruction and La­(OH)_3_ and MnOOH surface deposition from the adjacent solution. This observation supports the hypothesis that Ce acts as a redox mediator, facilitating Mn dissolution and importantly redeposition as active MnOOH species. This is consistent with the change in the seen improved Tafel slope at higher potentials. The other possible role of stabilizing intermediates is possible but does not explain the seen change in surface composition after restructuring and during OER seen by XPS and Raman spectroscopy. Finally, altering local pH or OH^–^ availability is also discounted, as activity enhancement was also seen in a high-pH environment (1 M).

After the OER, all Mn^2+^ disappears, and the oxidation states of Mn in LCM-0.1 are approximately 56.6% Mn^3+^ and 43.4% Mn^4+^, as shown in Table 6. This shows that the presence of CeO_2_ maintains an average lower Mn oxidation state in LM, resulting in formation of more active MnOOH and reduced formation of soluble Mn^6+/7+^.

For the O 1s spectra and their deconvoluted peaks, as shown in Figures S14e–f and 4e, the spectra reveal three distinct oxygen bonding peaks: metal–oxygen bonds (M–O) or lattice oxygen, metal hydroxide bonds (M–OH) or defective oxygen oxides, and absorbed water. The relative compositions of these peaks before and after the OER are listed in Table 6. After the OER, the composition of lattice oxygen peaks decreased from 55% to 38.8%, while the composition of M–OH peaks increased from 38.8% to 48.2%, along with an increase in the water peaks. These changes confirm the formation of MnOOH on the catalyst surface during the OER.^19,78,81^

### 3.3 Electrolyzer Performance

Figures 5 and 6 present the electrochemical performance of LaMnO_3_ (anode loading: 2.5 mg/cm^2^) and LCM-0.1 (anode loadings: 2.5 and 1.25 mg/cm^2^) in an anion exchange membrane (AEM) water electrolysis assembly. AEM water electrolyzers were fabricated using identical anode loadings (2.5 mg/cm^2^) of LaMnO_3_ (LM) and LCM-0.1 and tested in 0.1 and 1 M KOH under the different temperature conditions of 20, 40, and 60 °C, as shown in Figures 5 (a–i) and 6 (a–i) respectively.

**Figure 5:**
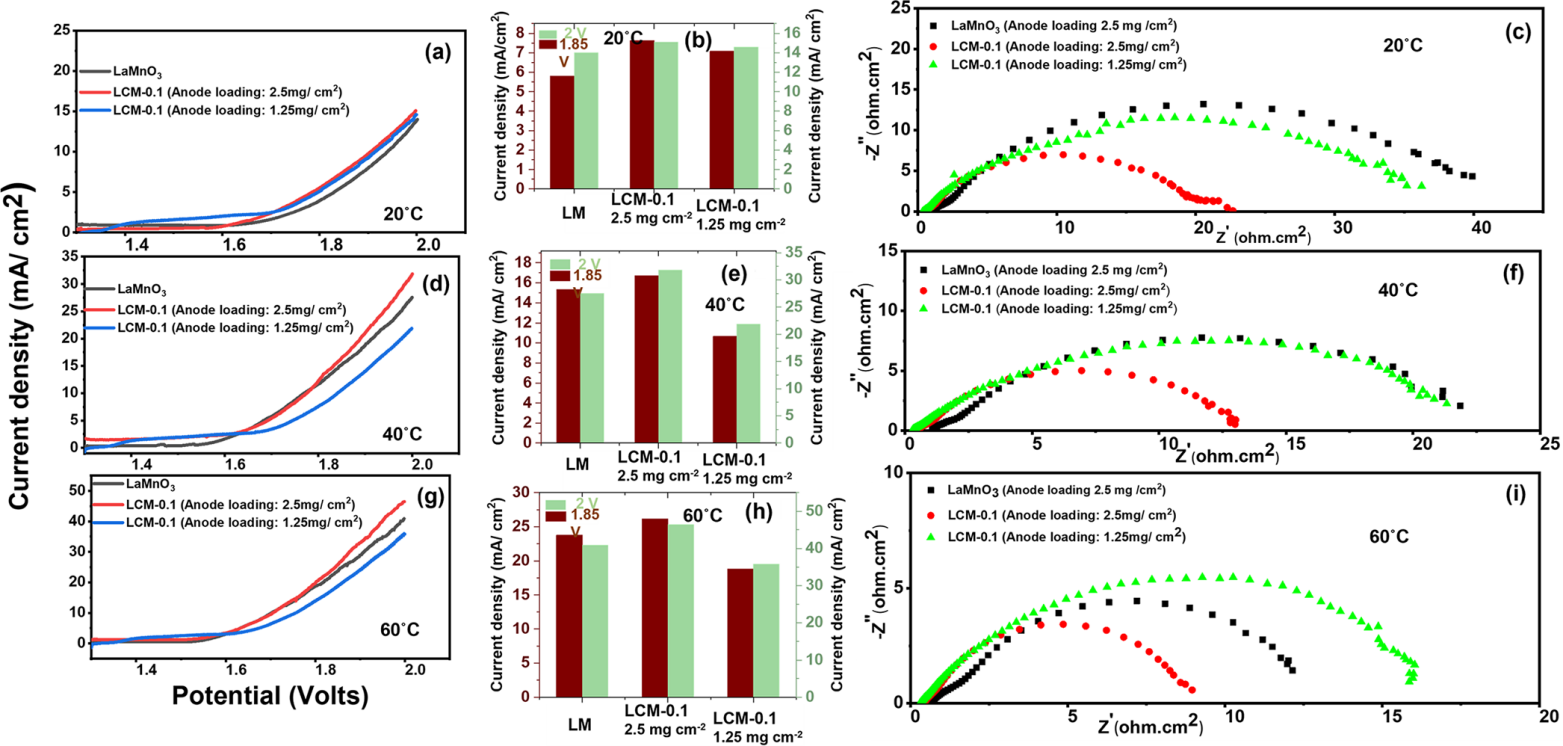
Water electrolysis in a 0.1 M alkaline solution: (a, d, g) polarization curves, (b, e, h) bar chart, and (c, f, i) Nyquist plot at 1.85 V of LM and LCM-0.1 with loadings of 2.5 and 1.25 mg/cm^2^ at 20, 40, and 60 °C.

**Figure 6:**
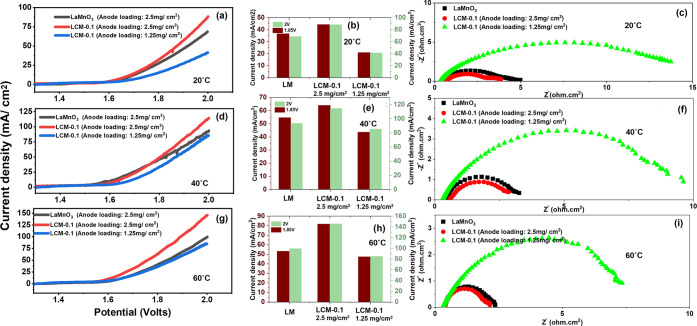
Water electrolysis in a 1 M alkaline solution: (a, d, g) polarization curves, (b, e, h) bar chart, and (c, f, i) Nyquist plot at 1.85 V of LM and LCM-0.1 with loadings of 2.5 and 1.25 mg/cm^2^ at 20, 40, and 60 °C.

The results indicate that the electrolyzer using LCM-0.1 with a 2.5 mg/cm^2^ loading exhibits significantly improved current densities at 1.85 and 2.0 V compared to the assemblies using either 2.5 mg/cm^2^ LM or 1.25 mg/cm^2^ LCM-0.1. Notably, with 0.1 M KOH at 60 °C, the LCM-0.1 electrode with 2.5 mg/cm^2^ loading achieves a maximum current density of 46.67 mA/cm^2^ at 2.0 V (see Figure 5h). Furthermore, the same LCM-0.1 configuration demonstrated a lower area-specific resistance (ASR) of 0.469 Ω·cm^2^ at 60 °C in 0.1 M KOH, compared to 0.688 Ω·cm^2^ for LM under identical conditions, as shown in Table S3.

Improved ASR values by increasing temperature can be regarded as one of the reasons for the observation of improved electrolyzer performance. Concurrently, as can be inferred from the impedance data, charge transfer resistance is one of the main factors, and it is reduced at higher temperatures. Interestingly, the data indicate that the inclusion of cerium oxide has resulted in a considerable reduction of ASR. This is surprising given the poor electronic conductivity of CeO_2_ but can be due to ceria acting as a OH^–^ conductor through its oxygen vacancies.

Evaluating electrocatalyst performance in an electrolyzer cell at a higher alkaline concentration (1 M KOH) resulted in an increase in the generated currents (Figure 6 (a–i)), where at 2 V, LaMnO_3_ achieved 68.59 mA/cm^2^ at 20 °C, as opposed to 88.28 mA/cm^2^ with ∼10 wt % cerium oxide inclusion (LCM-0.1) at the same temperature (20 °C). This was further improved to 93.57 mA/cm^2^ (40 °C) and 99.72 mA/cm^2^ (60 °C) for pure LaMnO_3_ and to 114.53 mA/cm^2^ (40 °C) and 145.79 mA/cm^2^ (60 °C) for LCM-0.1.

The main increase in current density can be attributed to the increase of catalyst activity or reduction in charge transfer resistance, as can be clearly seen in Nyquest plots in Figures 5(c,f, and i) and 6(c,f, and i), where the diameter of the semicircle in the spectra decreased when moving from LaMnO_3_ to LCM-0.1 (1.25 mg cm^–2^) to LCM-0.1 (2.5 mg cm^–2^), e.g., from 15 to 12 to 9 Ω cm^2^ in Figure 5i. Similarly, charge transfer resistance decreases with temperature increase from 20 to 60 °C (Figure 5c,f, and i) as well as with an increase in alkaline supporting electrolyte concentration at given conditions, e.g., from 9 to 2.5 ohm cm^2^ at 60 °C when moving from 0.1 to 1 M in LCM-0.1 (2.5 mg cm^–2^). Associated area-specific resistances are listed in Table S3. As can be seen at a higher concentration of 1 M, the ASR of LaMnO_3_ reduced by more than 50%, which could be expected, as the hydroxyl concentration is higher, e.g., from 1.097 to 0.395 ohm cm^2^ at 20 °C. The sample with a higher catalyst loading LCM-0.1 (2.5 vs 1.25 mg cm^–2^) also showed higher ASR, as expected due to higher OH^–^ ion resistivity in the thicker catalyst layer. With an increase in supporting electrolyte concentration and electrolyzer temperature, there was a minor difference in ASR between the three samples (1 M, 60 °C), but a significant difference in performance, confirming that charge transfer (catalyst activity) is the dominant factor in the change seen.

This improvement is in agreement with the results obtained from three-electrode cell experiments. It should be noted that performance variation in electrocatalysts used in water electrolyzer assembly can be due to differences in catalyst electrical conductivity at high current, reflected by electrocatalyst utilization and distribution of current on the electrodes, all of which are triggered by using a considerably higher amount of electrocatalyst loading (by about 10 times) compared to the half-cell experiments.

Comparing LCM-0.1 at two different catalyst loadings on the anode (2.5 and 1.25 mg cm^–2^) in the electrolyzer cell suggests a decrease in performance, especially at 40 and 60 °C, with the lowering of catalyst loading. Following the trend in ASRs, this observation can be explained by a trade-off among catalyst utilization, electrical conductivity, and surface area, and it highlights the importance of optimizing the electrocatalyst layer to minimize the energy losses occurring due to mass transport, ionic conductivity (ionomer), and electronic conductivity (LCM-0.1 electrocatalyst).

## 4 Conclusions

In conclusion, this study investigated synthesized lanthanum manganese perovskite oxide with various weight percentages of CeO_2_ content (up to 20%), revealing significant enhancements in electrocatalytic performance. The optimum performance was achieved at ∼10 wt % of CeO_2_ content, with OER activity improved by >22-fold at 1.9 V versus RHE as compared to that of the pure LaMnO_3_ perovskite, and beyond this optimal threshold, the inactive characteristics of CeO_2_ became predominant, underscoring the importance of CeO_2_ content integration. Electrochemical analyses highlighted the changes and restructuring of the catalyst during the OER testing and synergistic effects between the two oxide phases, enhancing electrocatalytic activity. The OER activity of LM and LCM-0.1 were tracked via in-operando Raman spectroscopy and ex-situ XPS spectroscopy, which evidenced reconstruction of the catalyst surface. The result confirms that electrocatalytic OER activity improvement has been attributed to restructuring of the catalyst surface to form the MnOOH structure in the presence of CeO_2_, with the loss of Mn and the coverage of the surface by inactive amorphous La­(OH)_3_ in the case of the CeO_2_-free LaMnO_3_ sample. Further, we have fabricated a water electrolyzer using the optimized LCM-0.1 and pure LM electrocatalysts with different anode catalyst loadings of 1.25 and 2.5 mg/cm^2^, and the device performance were evaluated in 0.1 and 1.0 M KOH with different temperatures of 20–60 °C. Notably, at 60 °C, the LCM-0.1 electrode with 2.5 mg/cm^2^ loading achieves a maximum current density of 145.79 mA/cm^2^ at 2.0 V with 1.0M KOH. Overall, these findings suggest that there is restructuring occurring on the perovskite surface during OER, and the active species remain transition metal oxyhydroxides. The incorporation of cerium oxide in perovskite-based systems is a promising strategy for enhancing oxygen evolution electrocatalytic activity in water electrolyzers by stabilizing the transition metal at a lower oxidation state during the OER, paving the way for more efficient energy conversion technologies.

## Supplementary Material



## Notes

Additional BET analysis; SEM and EDX images; Rietveld refinement graph of the electrocatalysts; XPS analysis; conversion of potential; iodometric titration reaction details; calculation of crystalline size; and comparation table of area-specific resistances (ASRs) of electrocatalysts (PDF)
