# Supplementary material for: Enhanced Oxygen Evolution Reaction Activity of a Cerium Oxide-Modified Lanthanum Manganese Oxide Perovskite Catalyst in an Anion Exchange Membrane Water Electrolyzer
Source: Energy Fuels. 2025 Oct 14;39(42):20593–608. doi: 10.1021/acs.energyfuels.5c03080 (PMC12557202; doi:10.1021/acs.energyfuels.5c03080)
Supplement: Supplementary file 1 [file ef5c03080_si_001.pdf]

Supporting Information

Enhanced Oxygen Evolution Reaction Activity of a Cerium Oxide Modified Lanthanum Manganese Oxide Perovskite Catalyst in an Anion Exchange Membrane Water Electrolyzer

Masoud Nouri [a], Jian Huang [a], Shanmugam Ramakrishnan [a], Gaurav Gupta [b], Stevin Pramana [a], Mohamed Mamlouk [a*]

[a] School of Engineering, Newcastle University, Newcastle upon Tyne, NE1 7RU, United Kingdom

[b] School of Engineering, Lancaster University, Lancaster, LA1 4YW, United Kingdom

*mohamed.mamlouk@newcastle.ac.uk

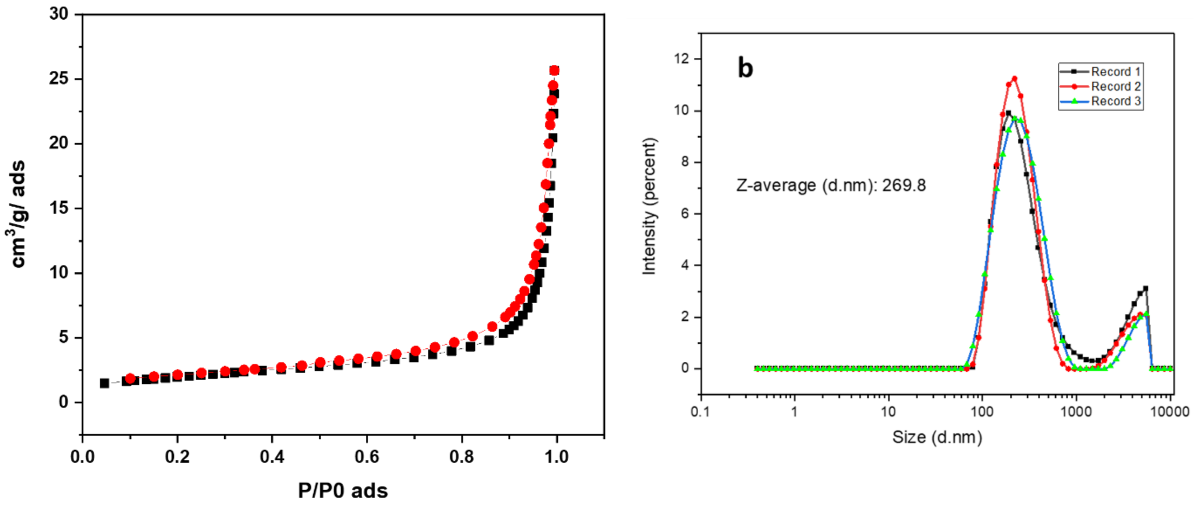

Figure S1. a) Obtained BET isotherm (adsorption and desorption curves in N_2 medium) for $LaMnO_3$ – 7.0350 m^2/g surface area; b) An example of the measured particle size distribution curve and average the samples were 269 nm of particle size.

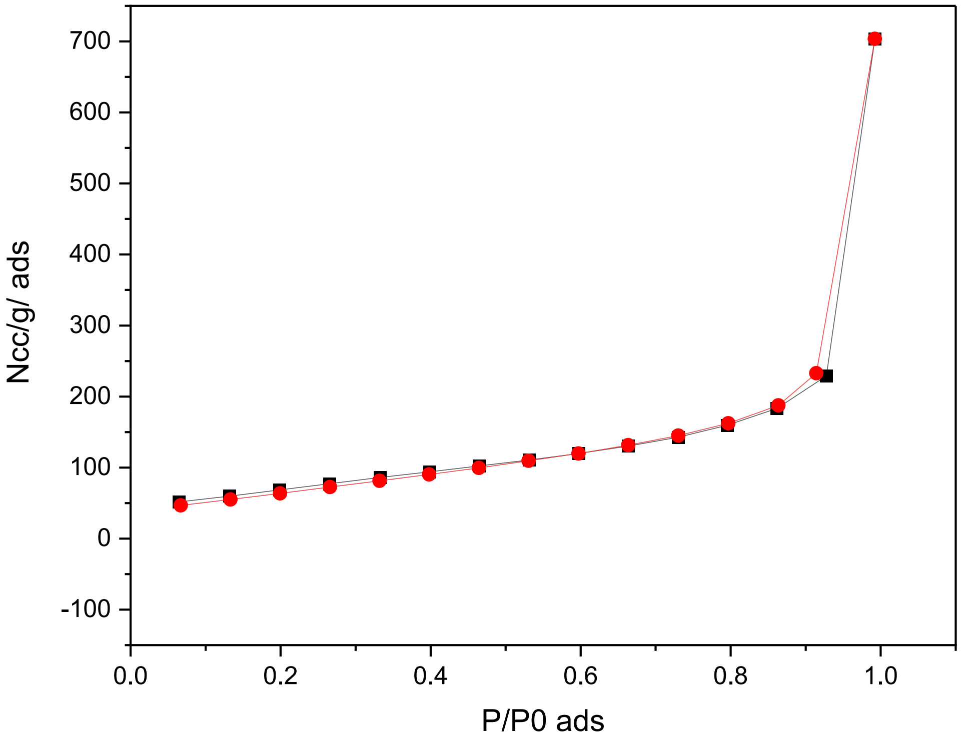

Figure S2. Obtained BET isotherm (adsorption and desorption curves in N_2 medium) for LCM-0.05 with BET surface area: 5.87 m^2/g

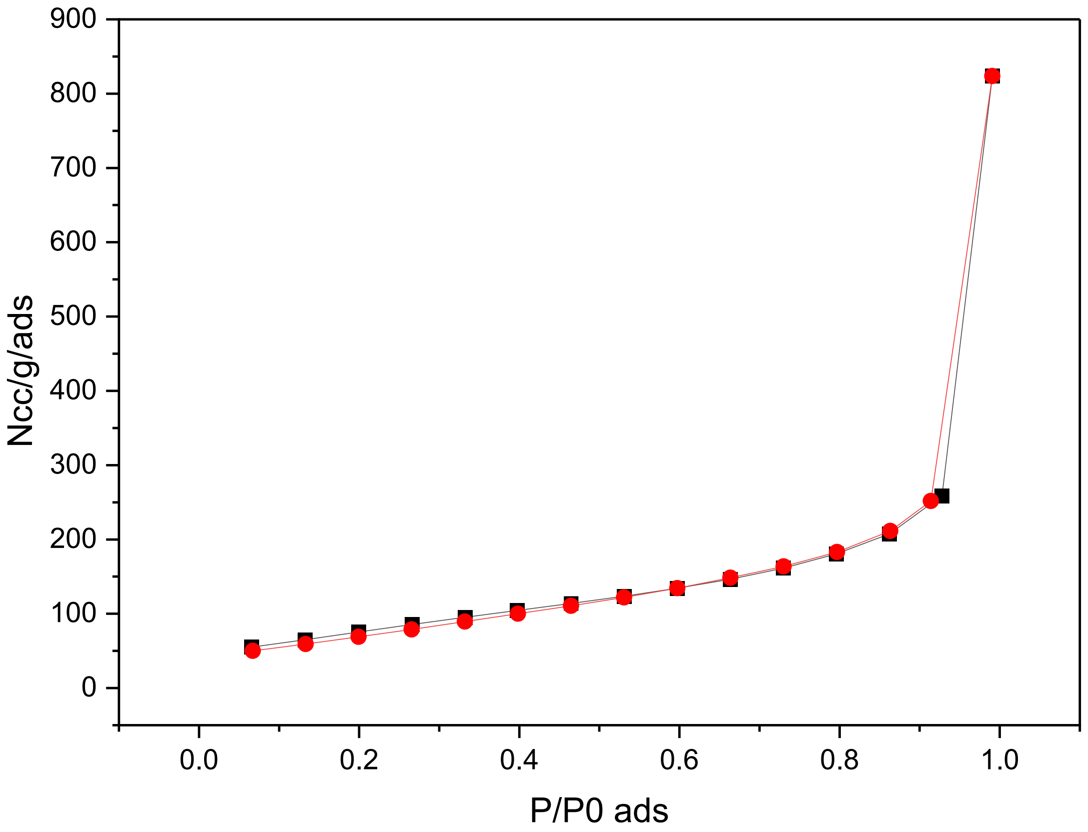

Figure S3. Obtained BET isotherm (adsorption and desorption curves in N_2 medium) for LCM-0.1
BET surface area: 6.46 m^2/g.

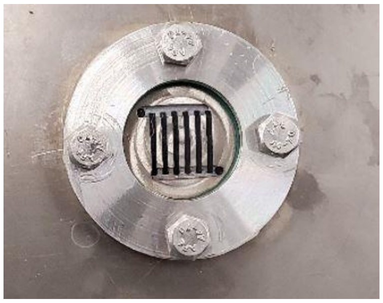

Figure S4. In operando T- cell for in-situ Raman spectroscopy

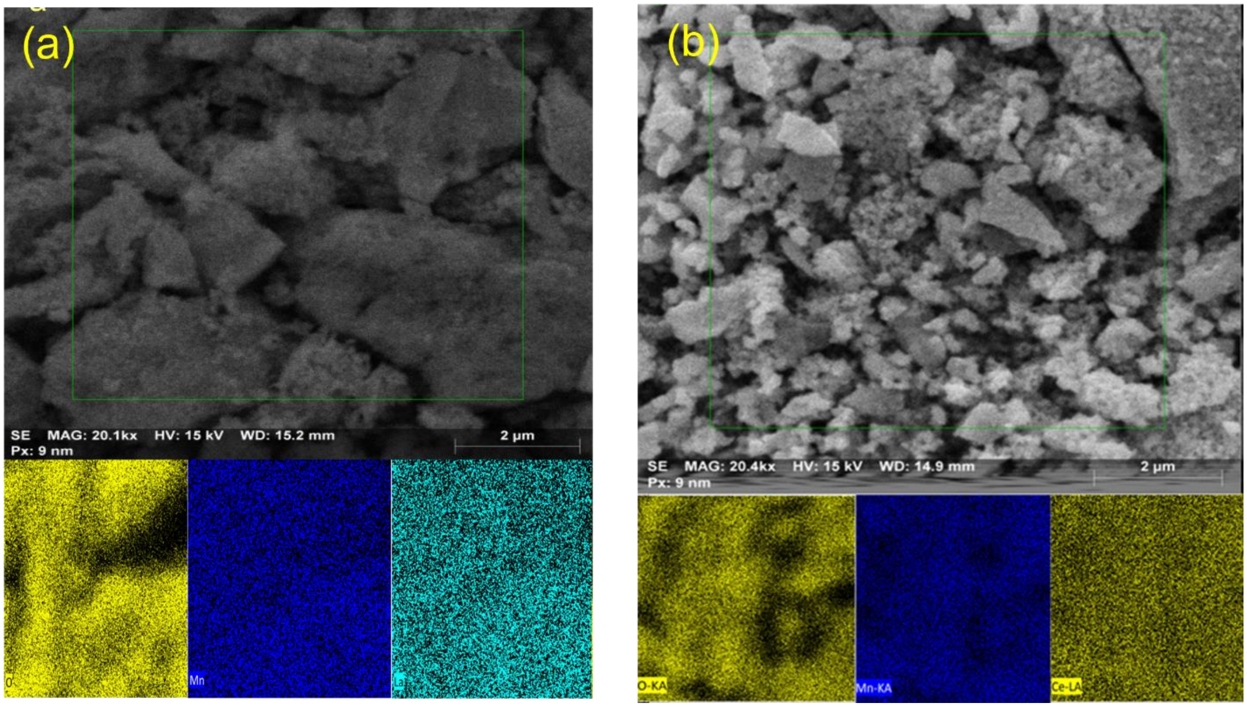

Figure S5. Scanning electron microscope (SEM) and associated elemental mapping by means of energy dispersive X-ray spectroscopy (EDX) on (a) pure $LaMnO_3$ and (b) CeO_2 – doped with $LaMnO_3$ (LCM-0.05)

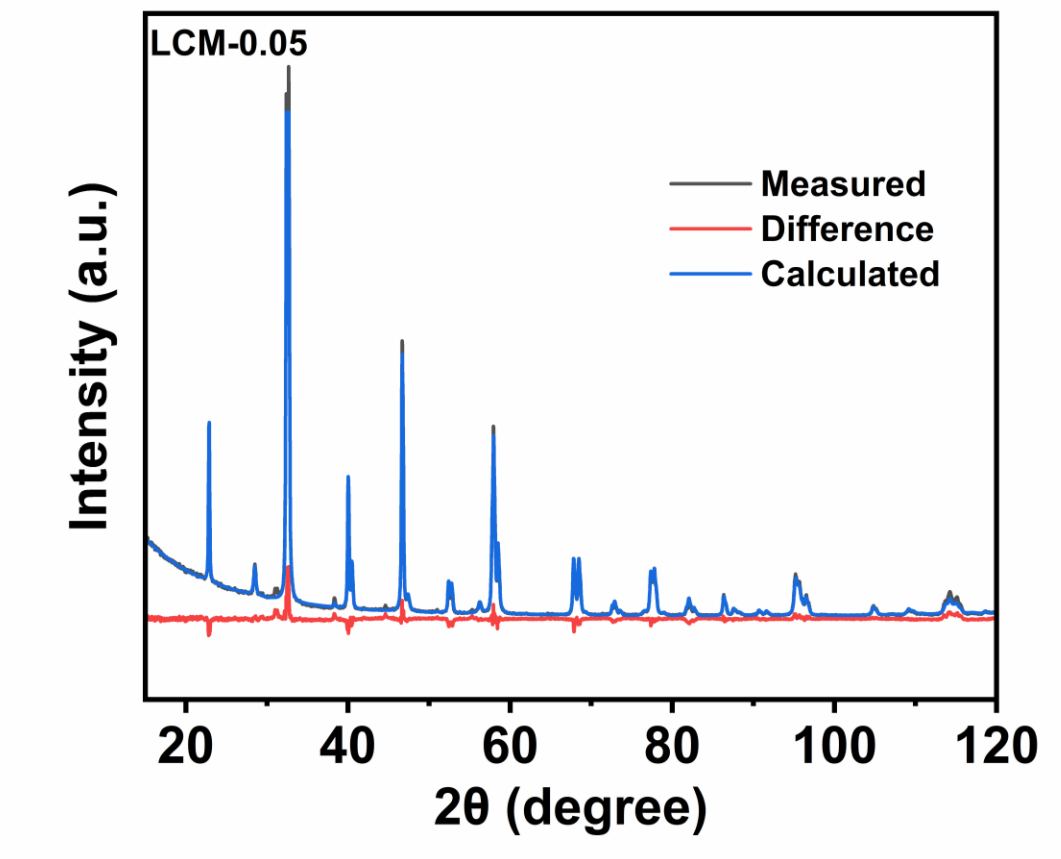

Figure S6. Rietveld refinement graph of lanthanum cerium manganese oxide with 5% intended cerium mixture (LCM-0.05) and Refinement shows the presence of 4.20wt% cerium oxide along with the perovskite phase (95.8%).

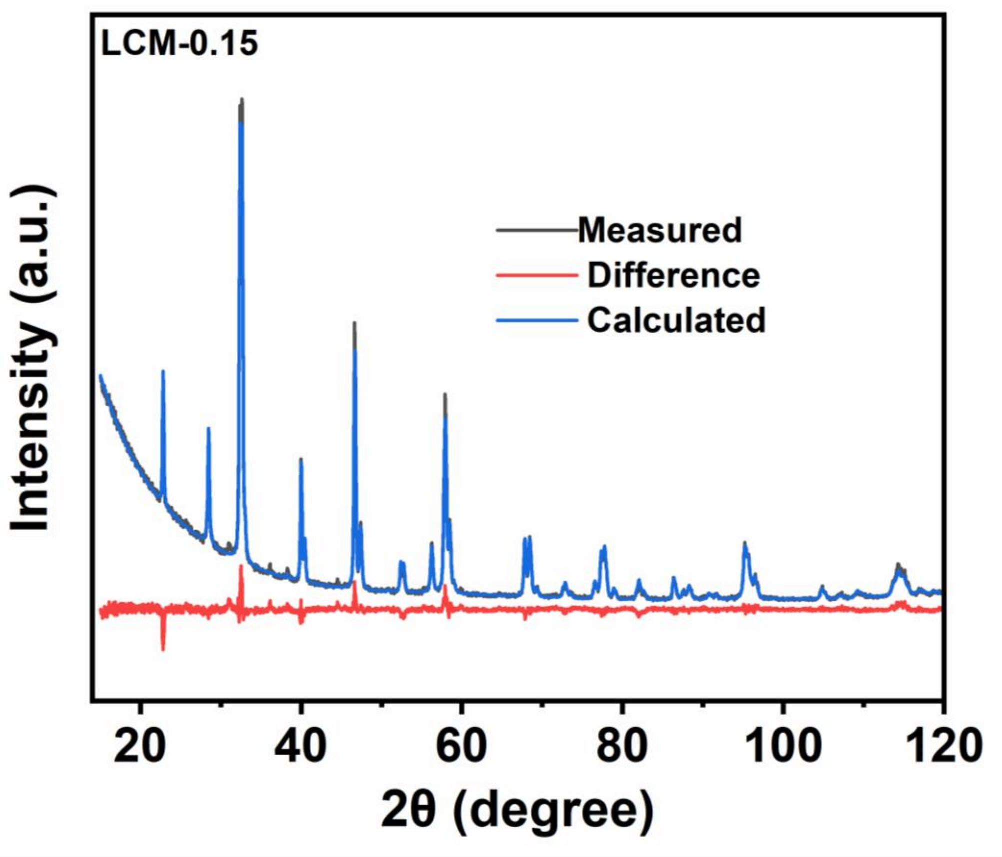

Figure S7. Rietveld refinement graph- lanthanum manganese cerium oxide (LCM-0.15) and and Refinement shows the presence of 12.8 wt% cerium oxide along with the perovskite phase (87.2%).

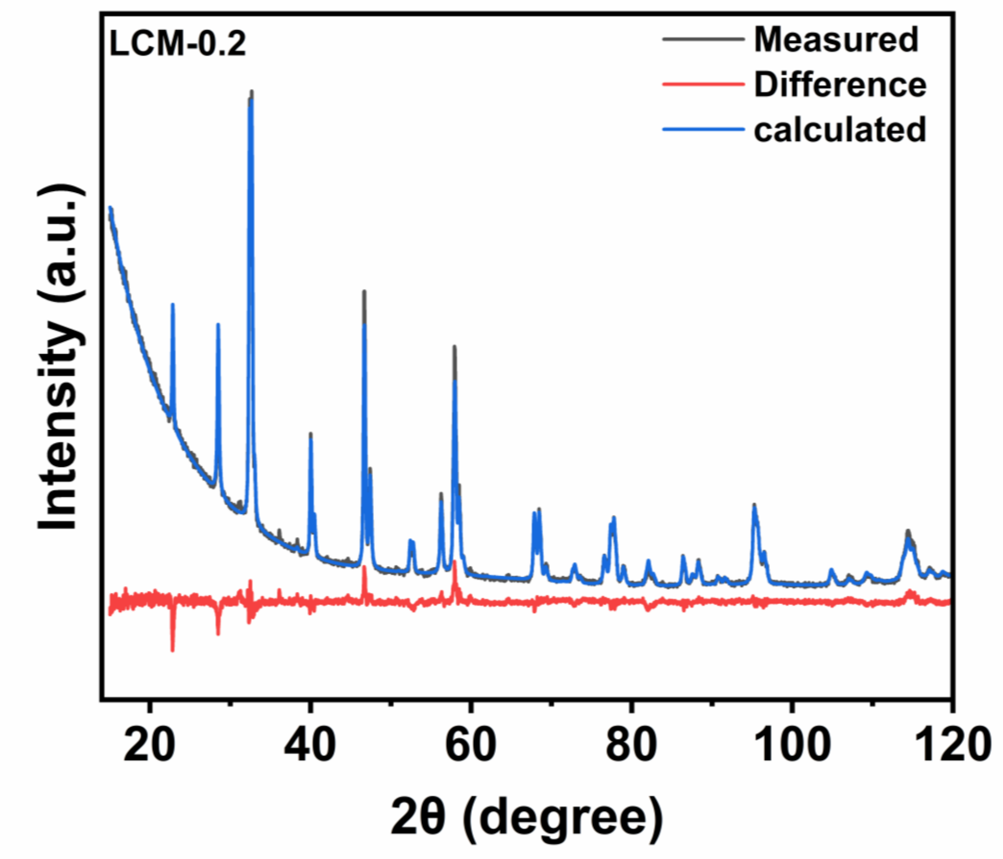

Figure S8. Rietveld refinement graph-lanthanum manganese cerium oxide (LCM-0.2) and and Refinement shows the presence of 19.8 % cerium oxide along with the perovskite phase (80.2%).

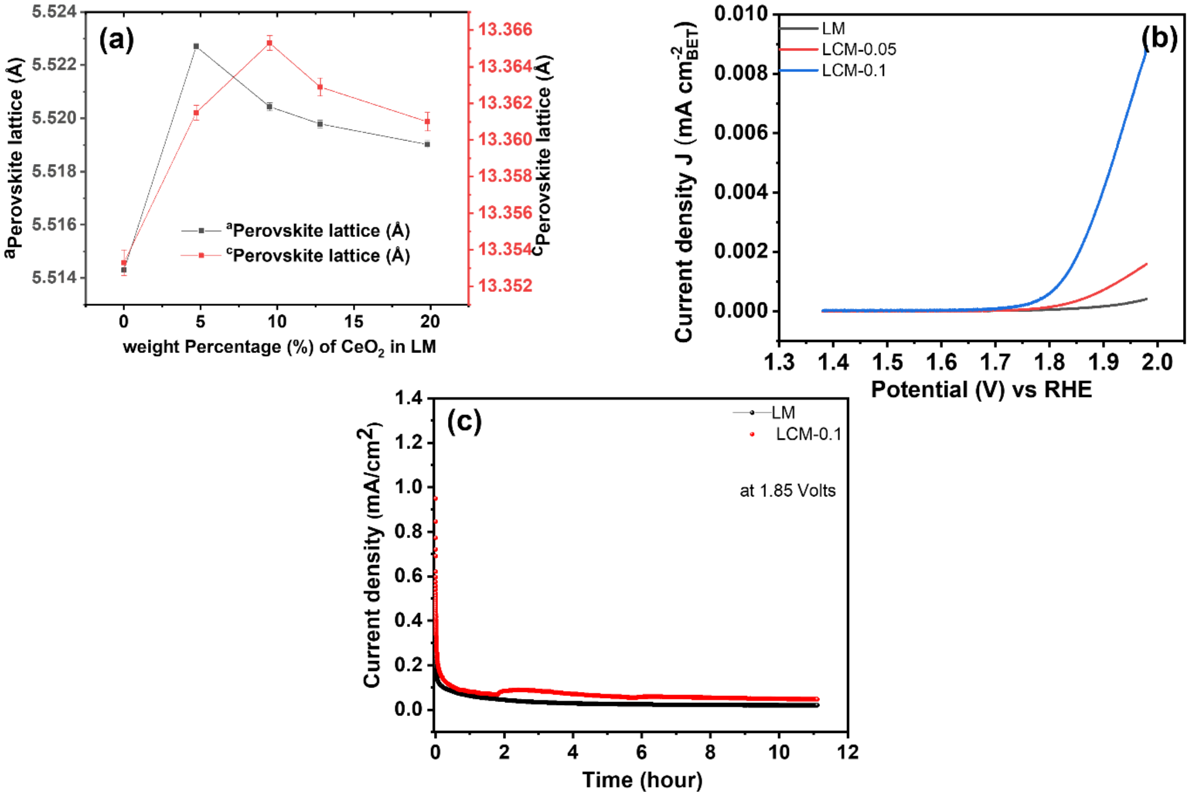

Figure S9. (a) Shows the scattering plot of weight % of CeO_2 in $LaMnO_3$ vs perovskite lattice parameter 'a,c', (b) OER activity curves with current density normalized to the BET specific surface area (intrinsic activity) and (c) Stability test; chronoamperometry test for LM and LCM-0.1with 11 hour at 1.85 vs RHE with 0.1M KOH.

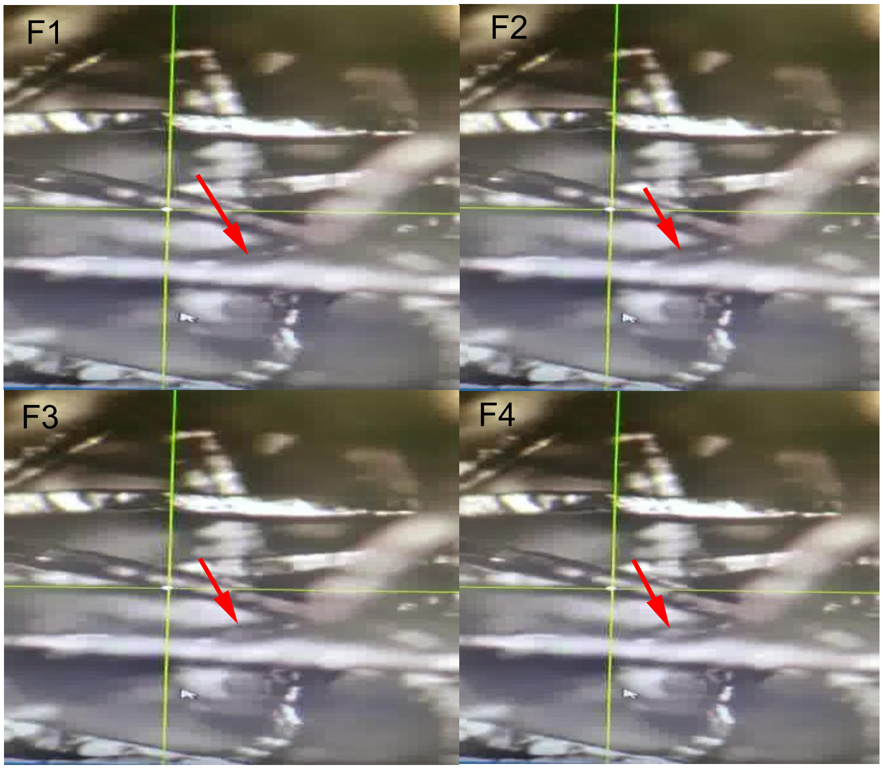

Figure S10. Frame-by-frame image extracted from the video capturing the evolved oxygen/ hydrogen molecules during the in-situ testing of electrocatalyst materials under Raman spectroscopy and red arrow follows the evolution of gaseous species.

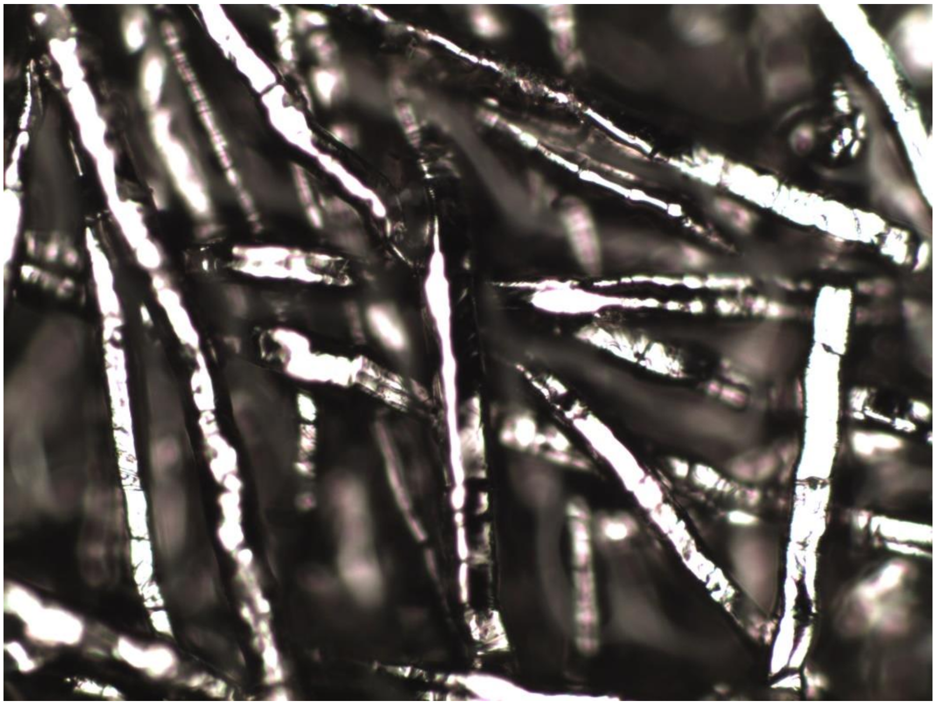

Figure S11. Microscopy image of LCM-0.1 coated catalyst on titanium observed under the microscope of Raman spectroscopy

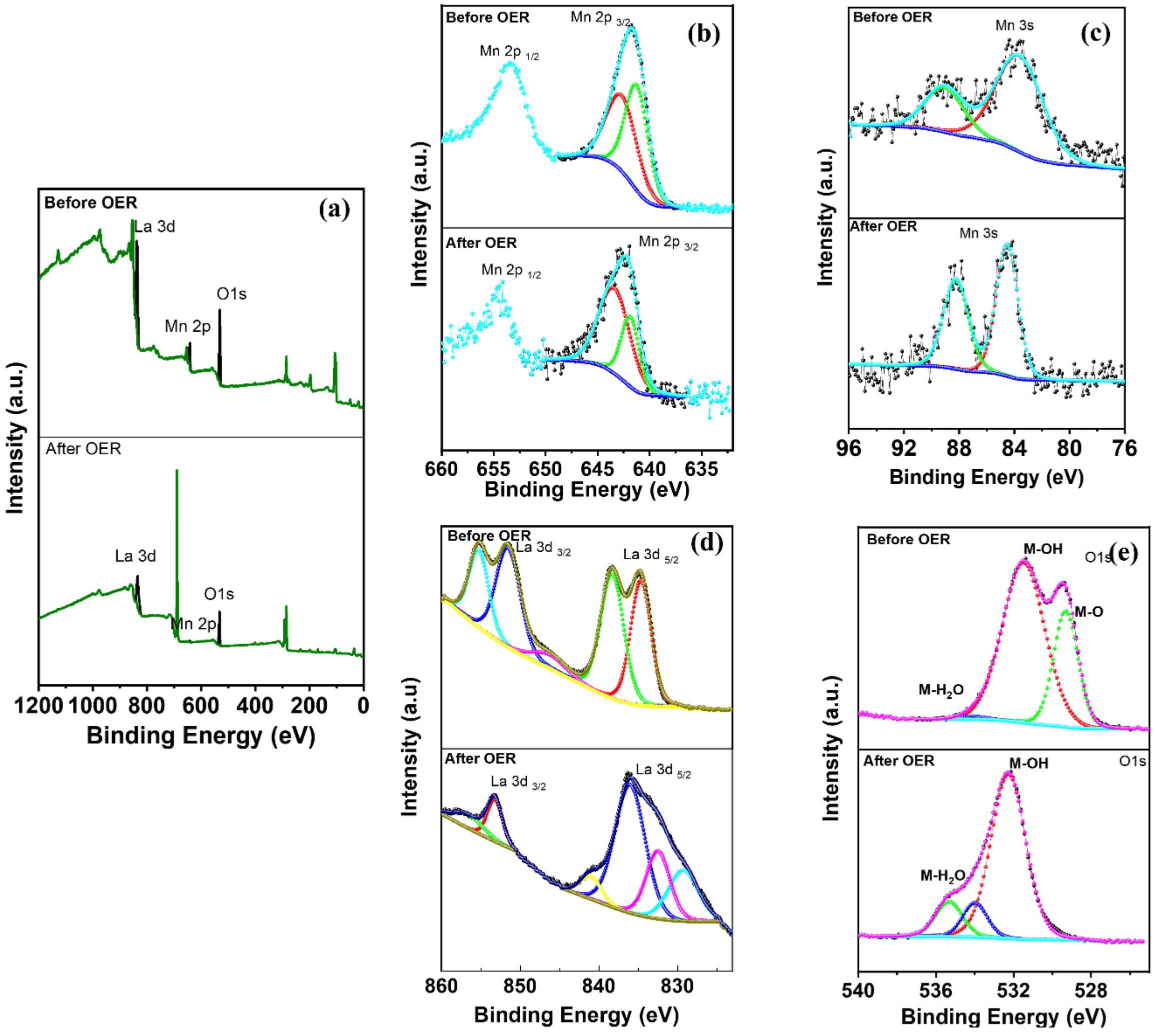

Figure S12. XPS spectrum of LM before and after OER test, (a) Survey spectrum, (b) Mn 2p, (c) Mn 3s, (d) La 3d and (e) O 1s.

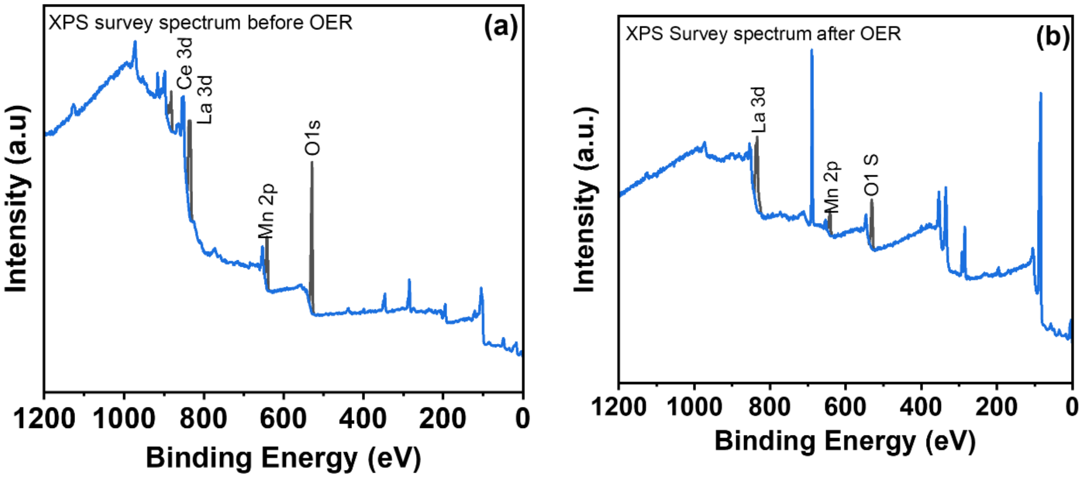

Figure S13. XPS survey spectrum of LCM-0.1 (a) before and (b) after OER test

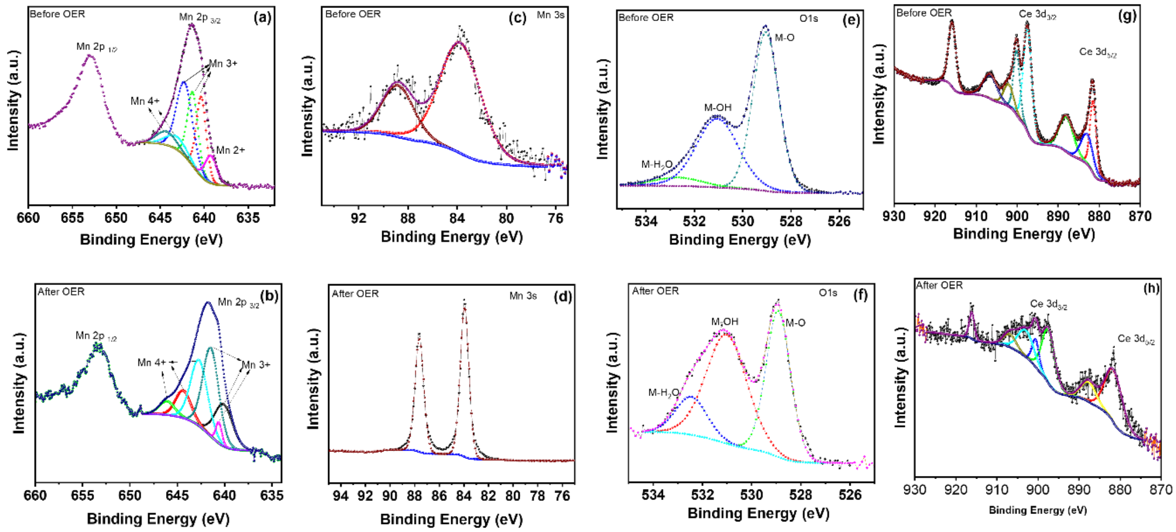

Figure S14. Deconvoluted XPS spectra of LCM-0.1 before and after OER test, (a-b) peaks of Mn 2p, (c-d) peaks of Mn 3s, (e-f) peaks of O1s and (g-h) peaks of Ce 3d.

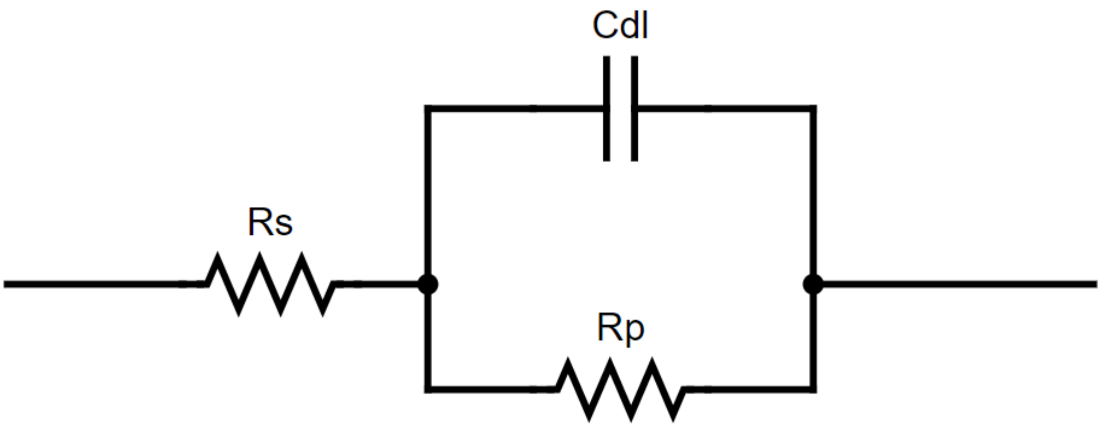

Figure S15. Equivalent circuit fit showing the components used to fit the impedance curve- R_s: solution resistance, R_p: polarisation resistance, C_{dl}: electrical double layer capacitance.

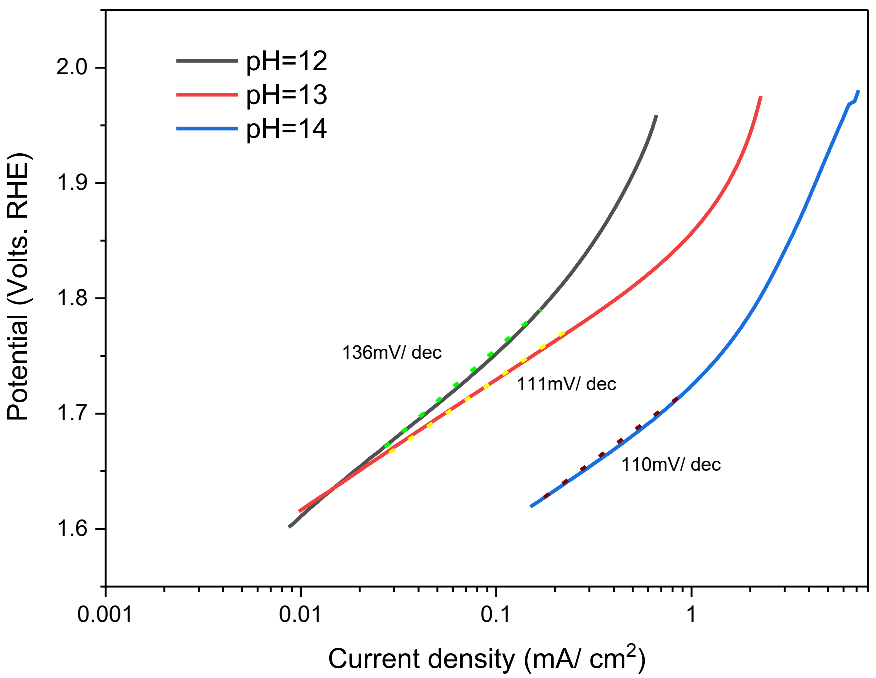

Figure S16. Tafel plot of LCM-0.1 after IR-correction at various pH levels from rotating ring disk electrode set-up at room temperature.

Conversion of potential Vs. Ag/ AgCl to potential Vs. RHE:

$$E\text{ (Volts vs RHE)} = E^{\circ}_{Ag/\,AgCl}\text{ vs SHE} + 0.059 * pH + E_{Ag/\,AgCl}$$

$$= 0.197 + 0.059 * 13 + E_{Ag/\,AgCl}$$

$$= 0.197 + 0.767 + E_{Ag/\,AgCl}$$

$$E\text{ (V vs RHE)} = 0.964 + E_{Ag/\,AgCl}$$

Iodometric titration reaction details:

$$Mn^{3+} + Cl^{-} \rightarrow Mn^{2+} + \frac{1}{2}Cl_2$$

$$Mn^{4+} + 2Cl^{-} \rightarrow Mn^{2+} + Cl_2$$

$$Cl_2 + 2I^{-} \rightarrow 2Cl^{-} + I_2$$

$$I_2 + 2S_2O_3^{2-} \rightarrow 2I^{-} + S_4O_6^{2-}$$

Calculation of crystalline size for cerium oxide from XRD data:

Scherrer's equation was used for crystalline size estimation (D) as follows:

$$D = \frac{K\lambda}{\beta\cos\theta}$$

$$R_{wp} = \sqrt{\frac{\sum w_m\left(Y_{o,m} - Y_{c,m}\right)^2}{\sum w_m {Y_{o,m}}^2}}$$

$$GOF = \sqrt{\frac{\sum w_m (Y_{o,m} - Y_{c,m})^2}{M - P}}$$

$Y_{o,m}$, $Y_{c,m}$, M and P are the observed data, calculated data, number of data points and number of parameters, respectively, whilst w_m is given by $1/\sigma(Y_{o,m})^2$ where $\sigma(Y_{o,m})$ is the error in $Y_{o,m}$

Where θ is the peak position, β is full width at half maximum (FWHM), K is a constant usually 0.94 for spherical crystals with cubic symmetry and λ is the wavelength of the x-ray beam used to obtain the data.

Table S1. Electrochemical data obtained from cyclic voltammetry and Tafel Extrapolation after IR correction

**Sample**	**Tafel slope (V/ decade)**	**I_0 ($A.cm^{-2}$)**	**I ($mA.cm^{-2}$) at 1.8 volts**	**I ($mA.cm^{-2}$) at 1.9 volts**
LM	0.187	3.50×10^{-12}	0.014	0.042
LCM-0.05	0.117	1.59×10^{-17}	0.032	0.149
LCM-0.1	0.109	2.07×10^{-18}	0.141	0.946
LCM-0.15	0.108	5.11×10^{-18}	0.200	0.857
LCM-0.2	0.116	4.90×10^{-17}	0.064	0.439

Table S2. Comparison of OER Performance with Recently Reported Perovskite Catalysts

S. No	Electrocatalyst	Electrolyte (KOH) M	Potential (V) @ Current density (mA cm^{-2})	Specific activity@ Potential (V) @ mA·cm$^{-2}_{BET}$	Tafel slope mV dec-1	Reference
1	LCM-0.1	0.1	1.8 @0.141<br>1.9 @ 0.9407	1.9 @0.082	109	This work
2	$LaMn_{0.2}Co_{0.8}O_3$	1	1.660 @ 10		57.95	[1]
3	$LaMn_{0.2}Co_{0.8}O_3$	0.1	1.65 @1<br>1.77 @5		100	[2]
4	$La_{0.6}Sr_{0.4}Co_{0.8}Fe_{0.2}O_{3-\delta}$ (LSCF)	0.1	1.566 @10		106.02	[3]
5	$La(Co_{0.55}Mn_{0.45})_{0.99}O_{3-\delta}$ NR/N-rGO	0.1	1.742 @ 10		-	[4]
6	$LaMn_{0.7}Co_{0.3}O_3$	0.1	1.820 @ 10			[5]
7	$LaCr_{0.2}Mn_{0.2}Fe_{0.2}Co_{0.2}Ni_{0.2}O_{3-\delta}$	0.1	1.68 @≈ 3.7		51	[6]
8	urea-assisted - $LaMnO_3$ (3.0U-LMO)	0.1	1.738 @1	-	-	[7]
9	$La_2NiO_{4+\delta}$	0.1	1.68 @4.53	1.68 @4.55	114	[8]
10	$La_3Ni_2O_{7-\delta}$	0.1	1.68 @5.17	1.68 @ 5.95	87	[8]

11	$La_4Ni_3O_{10-\delta}$	0.1	1.68 @7.75	1.68@8.18	77	8
12	$La_5Ni_4O_{13-\delta}$	0.1	1.68 @16.4	1.68@18.2	70	8

Table S3. Area specific resistances (ASRs) of electrocatalysts in 0.1 and 1M potassium hydroxide at various temperatures- alkaline electrolyser cell study

**Sample details**	**20°C (ohm.cm^2)**		**40°C (ohm.cm^2)**		**60°C (ohm.cm^2)**	
Electrolyte concentration (M KOH)	0.1	1	0.1	1	0.1	1
$LaMnO_3$ (loading:2.5mg/ cm^2)	1.097	0.395	1.075	0.414	0.688	0.352
LCM-0.1 (loading:2.5mg/ cm^2)	0.622	0.603	0.539	0.520	0.469	0.356
LCM-0.1 (loading: 1.25mg/ cm^2)	0.347	0.304	0.315	0.312	0.290	0.286

References:

(1) Vazhayil, A.; Thomas, J.; Thomas, N. Cobalt doping in $LaMnO_3$ perovskite catalysts – B site optimization by solution combustion for oxygen evolution reaction. *J. Electroanal. Chem.* **2022**, *918*, 116426.

(2) Flores-Lasluisa, J. X.; Huerta, F.; Cazorla-Amorós, D.; Morallon, E. Carbon Material and Cobalt-Substitution Effects in the Electrochemical Behavior of $LaMnO_3$ for ORR and OER. *Nanomaterials* **2020**, *10* (12), 2394.

(3) Fu, C.; Ma, Q.; Wu, Q.; Yuan, Z.; Wu, Z.; He, J.; Li, X. Boosting bifunctional catalytic activity of perovskite nanoparticles for rechargeable Zn-air batteries. *Mater. Chem. Phys.* **2022**, *290*, 126557.

(4) Ge, X.; Goh, F. W. T.; Li, B.; Hor, T. S. A.; Zhang, J.; Xiao, P.; Wang, X.; Zong, Y.; Liu, Z. Efficient and durable oxygen reduction and evolution of a hydrothermally synthesized $La(Co_{0.55}Mn_{0.45})_{0.99}O_{3-\delta}$ nanorod/graphene hybrid in alkaline media. *Nanoscale* **2015**, *7* (19), 9046-9054.

(5) Liu, X.; Gong, H.; Wang, T.; Guo, H.; Song, L.; Xia, W.; Gao, B.; Jiang, Z.; Feng, L.; He, J. Cobalt-Doped Perovskite-Type Oxide $LaMnO_3$ as Bifunctional Oxygen Catalysts for Hybrid Lithium–Oxygen Batteries. *Chem. Asian J* **2018**, *13* (5), 528-535.

(6) Kante, M. V.; Weber, M. L.; Ni, S.; van den Bosch, I. C. G.; van der Minne, E.; Heymann, L.; Falling, L. J.; Gauquelin, N.; Tsvetanova, M.; Cunha, D. M.; et al. A High-Entropy Oxide as High-Activity Electrocatalyst for Water Oxidation. *ACS Nano* **2023**, *17* (6), 5329-5339.

(7) Luo, K.; Zheng, Q.; Yu, Y.; Wang, C.; Jiang, S.; Zhang, H.; Liu, Y.; Guo, Y. Urea-Assisted Sol-Gel Synthesis of $LaMnO_3$ Perovskite with Accelerated Catalytic Activity for Application in Zn-Air Battery. *Batteries* **2023**, *9* (2), 90.

(8) Choi, S. R.; Lee, J.-I.; Park, H.; Lee, S. W.; Kim, D. Y.; An, W. Y.; Kim, J. H.; Kim, J.; Cho, H.-S.; Park, J.-Y. Multiple perovskite layered lanthanum nickelate Ruddlesden-Popper systems as highly active bifunctional oxygen catalysts. *Chem. Eng. J.* **2021**, *409*, 128226.
